# Supplementary material for: Functionality of chimeric TssA proteins in the type VI secretion system reveals sheath docking specificity within their N-terminal domains
Source: Nat Commun. 2024 May 20;15:4283. doi: 10.1038/s41467-024-48487-8 (PMC11106082; doi:10.1038/s41467-024-48487-8)
Supplement: Supplementary file 1 — Supplementary information [file 41467_2024_48487_MOESM1_ESM.pdf]

# SUPPLEMENTARY INFORMATION

## Functionality of chimeric TssA proteins in the type VI secretion system reveals sheath docking specificity within their N-terminal domains

**Selina Fecht[1], Patricia Paracuellos[1], Sujatha Subramoni[2], Casandra Ai Zhu Tan[2], Aravindan Ilangovan[3], Tiago R. D. Costa[1] and Alain Filloux[1,2*]**

[1]CBRB Centre for Bacterial Resistance Biology, Department of Life Sciences, Imperial College London, London, SW7 2AZ, UK

[2]Singapore Centre for Environmental Life Sciences Engineering, Nanyang Technological University, Singapore, 637551

[3]School of Biological and Behavioural Sciences, Queen Mary University of London, E1 4NS, UK

*Correspondence and request for materials should be addressed to A.F. (email: a.filloux@ntu.edu.sg)

## Supplementary Figures

**TssA1**

**1** MLDVPVLLAAVSPDSPCGDDLEYDAAFLELERIAQGQPERQMGDAVLPAEPPEWPRVRAL
**61** ASELFGRSKDLRVANLLLQSNVALDGLDGLADGLLLVRELLGQYWDGVYPLLDADDDNDP
**121** TFRINALTGLVAEPLLQLVWAIPLVRSRAFGPVNLRAALNAAGLQRFASETLSPEQIAGA
**181** FADADADALAATRRALDGAQEHALAIESGVAERVGSAQGLDLGPLRQLLRQALQVFDLYG
**241** PQGAGESLAPGAEAVADEQVGAAPVAAVAAPAPRASGEIANREDVLRQLDRLLEYYVRHE
**301** PSSPVPVLLKRAKTLVTADFAEIVRNLIPDGISQFETLRGPESE

Nt1 domain

C-terminal domain

**TssA2**

**1** MTYSSKLSSHYLELAEQPISKDVFAGEDVRFSSEYEALENELGKALSLHENGQTDWLKIL
**61** ENSEALLRAQSKDLRVAAWLTWALYQRESFPGLLAGLGLLERLCSRHWVEIHPLKARTRA
**121** AAIAWLVPRLEQALNENVPIKEQLPLFRRLAEHLEKLDTALTAQLGDDAPLLLPISRRLS
**181** GMLQRAADNQPEPGVVGAVVAQVKQAASQLLAPGAPIDNERDAHKAMRAQQEAARPLCAW
**241** WLKQKATDLRALRLNRTMLWLPIESMPERNAEQVTALRGVPADKLKSYQERFAQGLYADL
**301** LVELEASLARAPFWFDGQRLVWECLQGLNAEQAMREVEMHFALLLQRLPGLVELRFHDGS
**361** AFADAATRGWISAHVMPHLQNDSAPRKVETVALQAEWDVALDEVQPVLRKDGLKAAVQVL
**421** KQGMKRAHGGRARFFWQLSLARLCFLAKKYELAKTQLESLDHQLHESGLHAWEPDLALDV
**481** LHLLHSCCELLPQNHAVRERKEDIYRRLCHLDLEVVLE

Nt1 domain

Nt2 domain

C-terminal domain

**TssA3**

**1** MTLPLSGNALSLEVLLEPIDPGQACGPSLRYDPDYDRLRELRREDDSSLPTGVWQAEAKR
**61** ADWAAVEQLASELLQRRSKDLMLAAWLGEAWLQRGGLGGLQRALVLLAELCERYPEEVHP
**121** QAQDGDQSWRVPPIDWLLRRYAELLHTRLPLMGQGAFAEITLYAWQRLQRQQVASGDSKS
**181** AKAALEAAQLQQKKLDEALRAEPLVQWQRKQASLLACQQQLLRLEQWCDRCLGELAPSCQ
**241** PLREVIAQWLALLKEFIAMHPQAPLSEEQPPVAEADASEGDTDGEESVPASAPSGPAGAP
**301** TSREDAYRQLLLIADYLARTEPHSPVPYLIKRAVEWGNKPLSELLAELINADSEARRVWS
**361** LLGVLP

Nt1 domain

C-terminal domain

**Supplementary Fig. 1** Domain organisation of *P. aeruginosa* TssA proteins. **a** Annotation of the Nt1 (yellow) and C-terminal (red) domains in the TssA1 primary sequence. **b** Annotation of the Nt1 (yellow), Nt2 (purple) and C-terminal (blue) domains in the TssA2 primary sequence. **c** Annotation of the Nt1 (yellow) and C-terminal (red) domains in the TssA3 primary sequence.

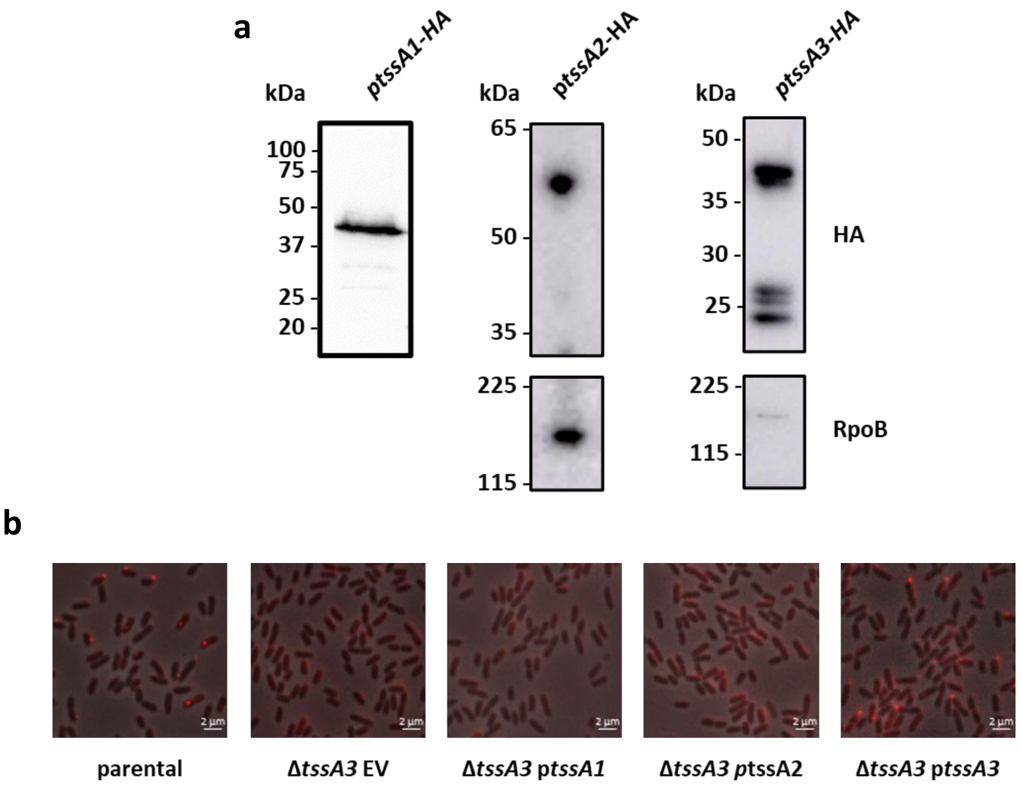


**Supplementary Fig. 2** Functional specificity of TssA proteins. **a** Expression of TssA proteins *in trans* in *P. aeruginosa*. TssA proteins were tagged at the C-terminal end with hemagglutinin (HA) and expressed from the pBBR1MCS4 vector in the PAO1Δ*retS* background for TssA1 and the PAO1Δ*rsmA* background for TssA2 and TssA3. Western blot analysis was carried out of HA-tagged chimeric proteins, with RpoB used as a loading control. **b** Functional specificity of TssA3 for the H3-T6SS. Representative images of TssB3-mScarlet sheath formation in the PAO1Δ*rsmA tssB3-mScarlet* background with cells expressing either wildtype *tssA3* in the parental strain, or with deletion of *tssA3* and either EV or *tssA1*, *tssA2* or *tssA3* expressed from pBBR1MCS4. Scale bar represents 2 µm.

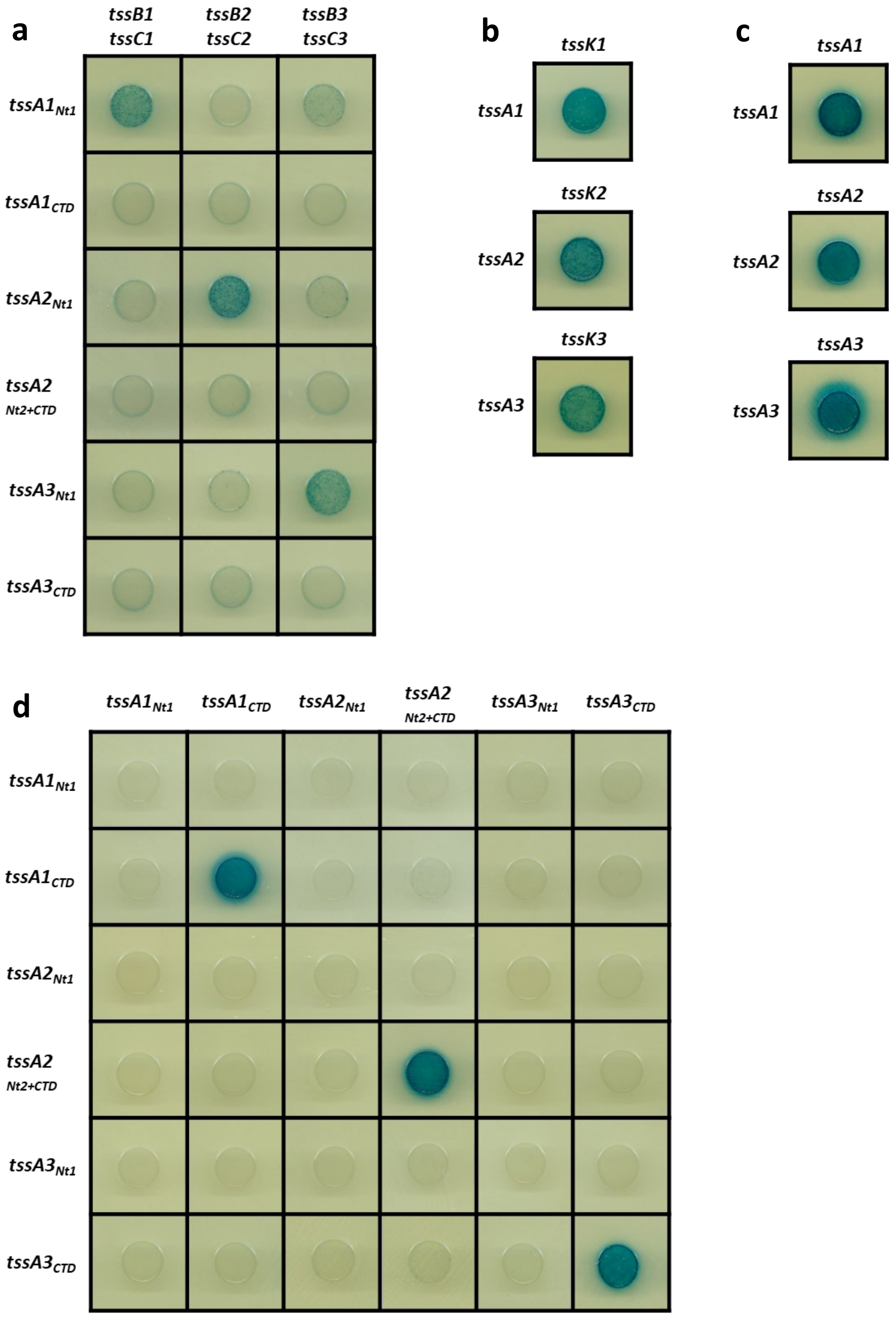


**Supplementary Fig. 3** Interactions of TssA proteins and domains. **a** Expanded view of Fig. 3b, BTH interactions between each TssA domain with each sheath complex. **b** BTH interactions of each TssA protein with the cognate TssK protein. **c** BTH self-interactions of TssA proteins. **d** BTH interactions between each TssA domain.

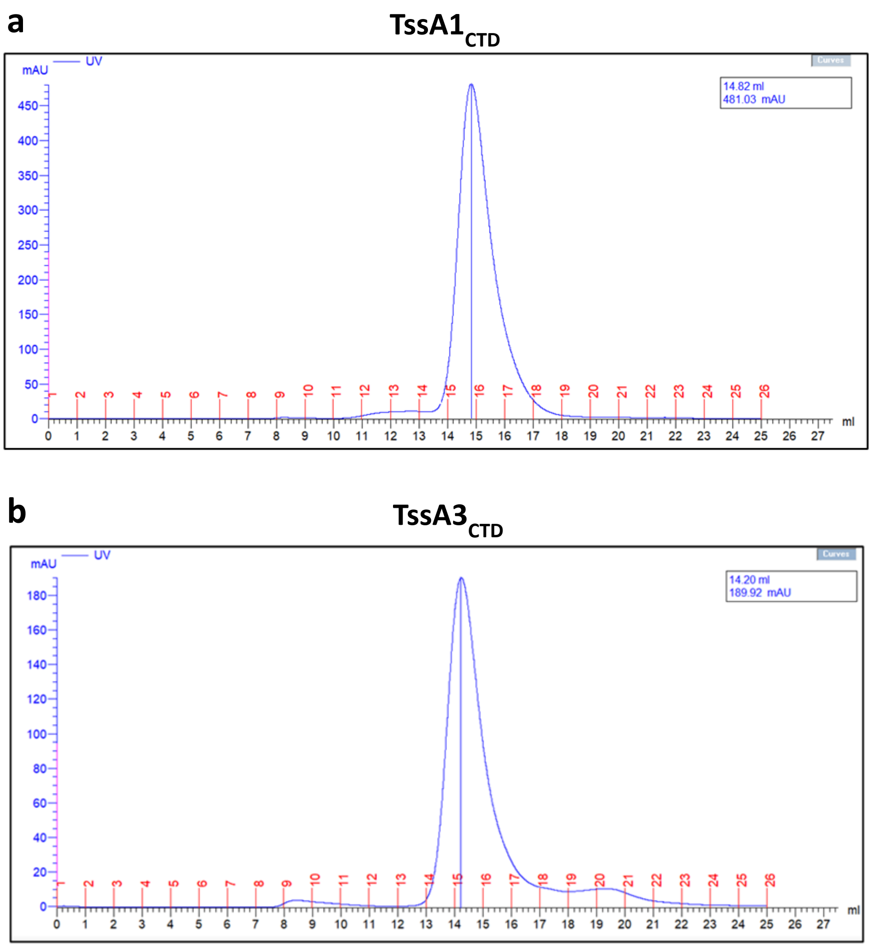


**Supplementary Fig. 4** Purification of TssA1 and TssA3 CTDs. **a** Size exclusion chromatography elution profile for TssA1 CTD. **b** Size exclusion chromatography elution profile for TssA3 C-terminal domain.

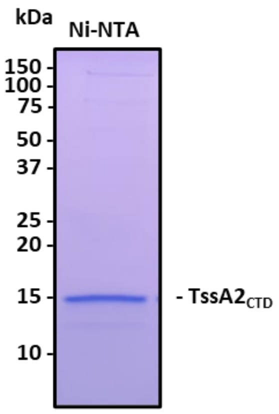


**Supplementary Fig. 5** Purification of TssA2 CTD. SDS-PAGE of the purified TssA2 CTD after Ni-NTA affinity chromatography.

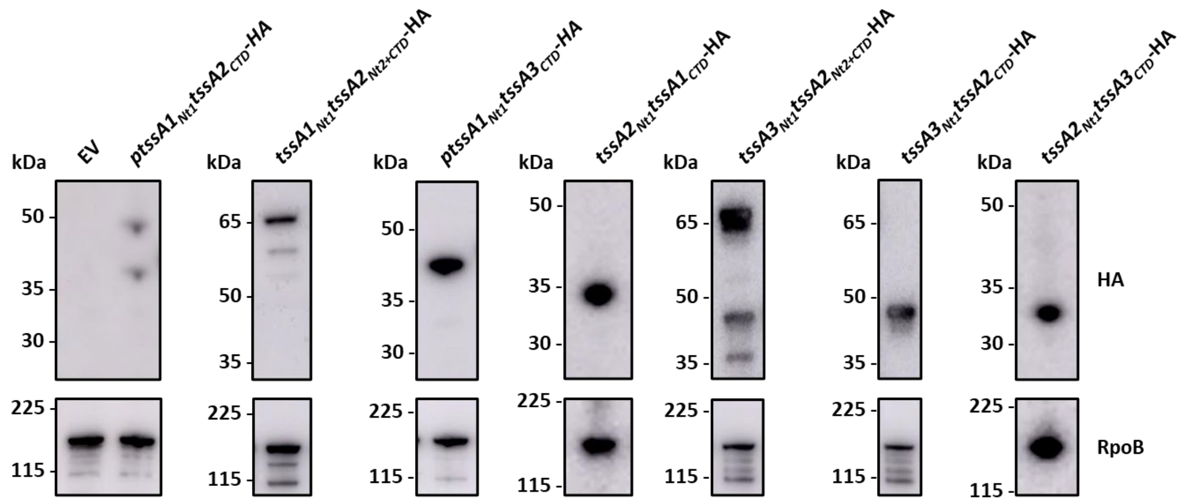


**Supplementary Fig. 6** Expression of chimeric TssA proteins. To detect their production, chimeric TssA proteins were tagged at the C-terminal end with hemagglutinin (HA) and expressed from the pBBR1MCS4 vector in *P. aeruginosa*. Western blot analysis was carried out of HA-tagged chimeric proteins, with RpoB used as a loading control. **a** Expression of *P. aeruginosa* chimeric TssA proteins. Production of TssA1$_{Nt1}$-domain containing chimeric proteins was assessed in the PAO1*ΔretS* background, while production of TssA2$_{Nt1}$- and TssA3$_{Nt1}$-domain containing chimeric proteins was assessed in the PAO1Δ*rsmA* background. A HA-tagged *tssA3$_{Nt1}$-tssA1$_{CTD}$* construct could not be generated, however the activity of this chimeric protein in the H3-T6SS suggests that this chimeric protein is produced and stable.

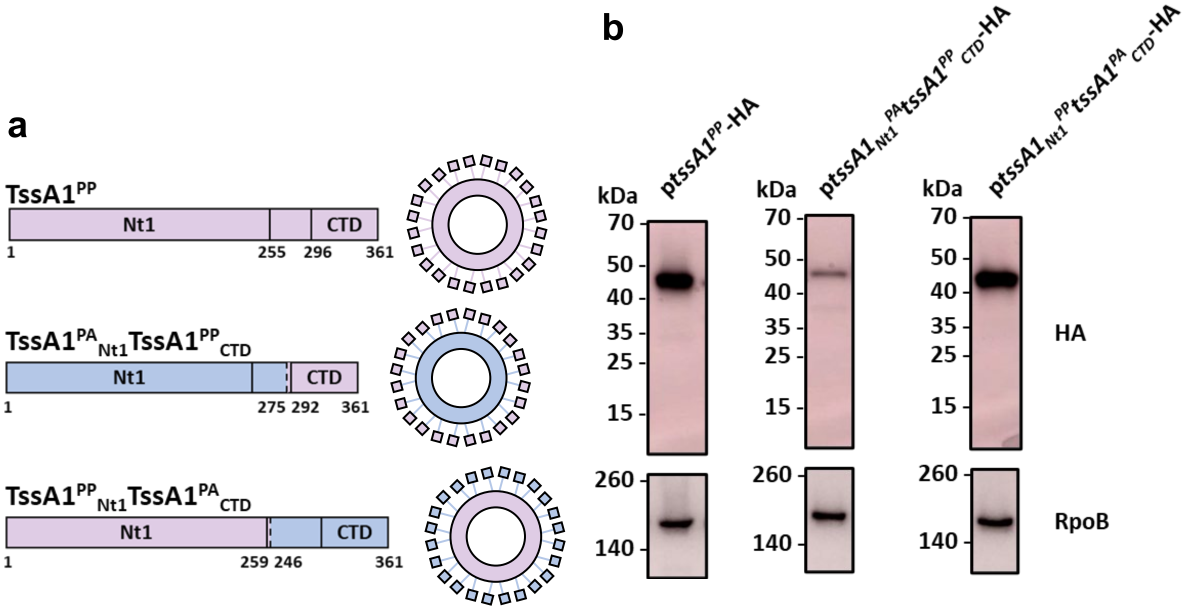


**Supplementary Fig. 7** Cross-species chimeric TssA proteins. **a** Schematic of *P. putida* wildtype and chimeric TssA proteins, with domains switched between *P. aeruginosa* TssA1 (TssA1$^{PA}$) and *P. putida* TssA1 (TssA1$^{PP}$). **b** Expression of cross-species *P. aeruginosa-P. putida* chimeric TssA proteins. Production of TssA1$^{PP}$ and cross-species chimeric TssA proteins was assessed in the PAO1*ΔretS* background.

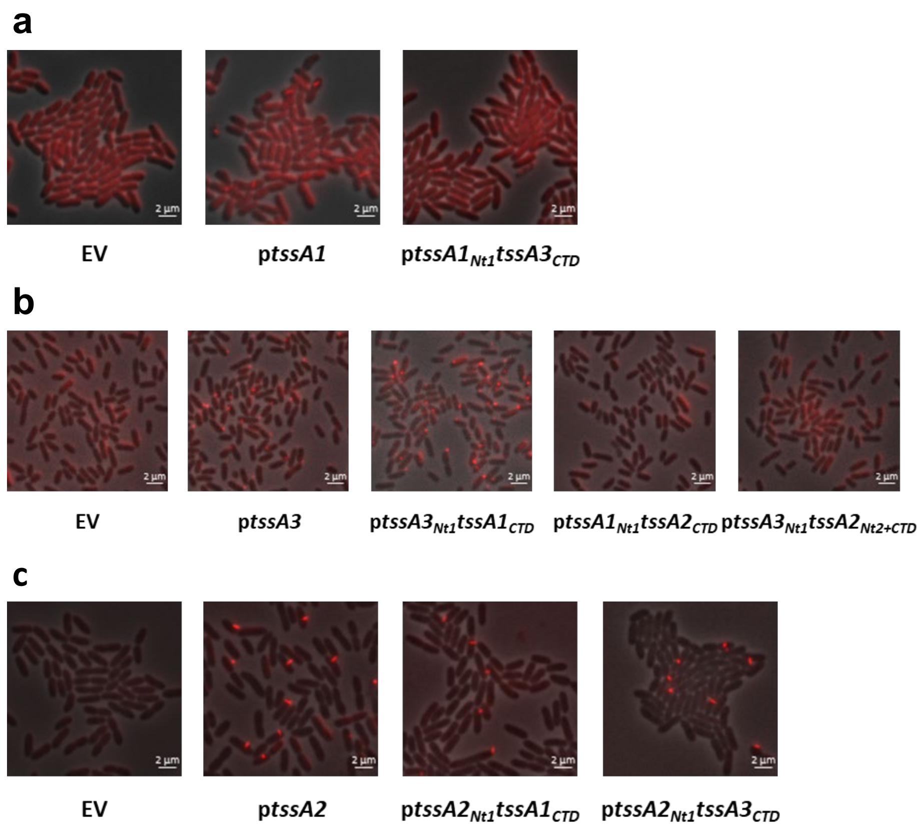


**Supplementary Fig. 8** Assessment of functionality of chimeric TssA proteins by fluorescence microscopy. To assess complementation of the *tssA* deletions, the pBBR1MCS4 vector was introduced either as empty vector (EV), encoding a wildtype *tssA* (p*tssA*) or chimeric *tssAs*. **A** Representative images of TssB1-mScarlet sheath formation in the PAO1Δ*retS*Δ*tssA1 tssB1-mScarlet* background, with either EV or wildtype or chimeric *tssA*s. **b** Representative images of TssB3-mScarlet sheath formation in the PAO1Δ*rsmA*Δ*tssA3 tssB3-mScarlet* background, with either EV or wildtype or chimeric *tssA*s. **c** Representative images of TssB2-mScarlet sheath formation in the PAO1Δ*rsmA*Δ*tssA2 tssB2-mScarlet* background, with either EV or wildtype or chimeric *tssA*s. Scale bar represents 2 μm.

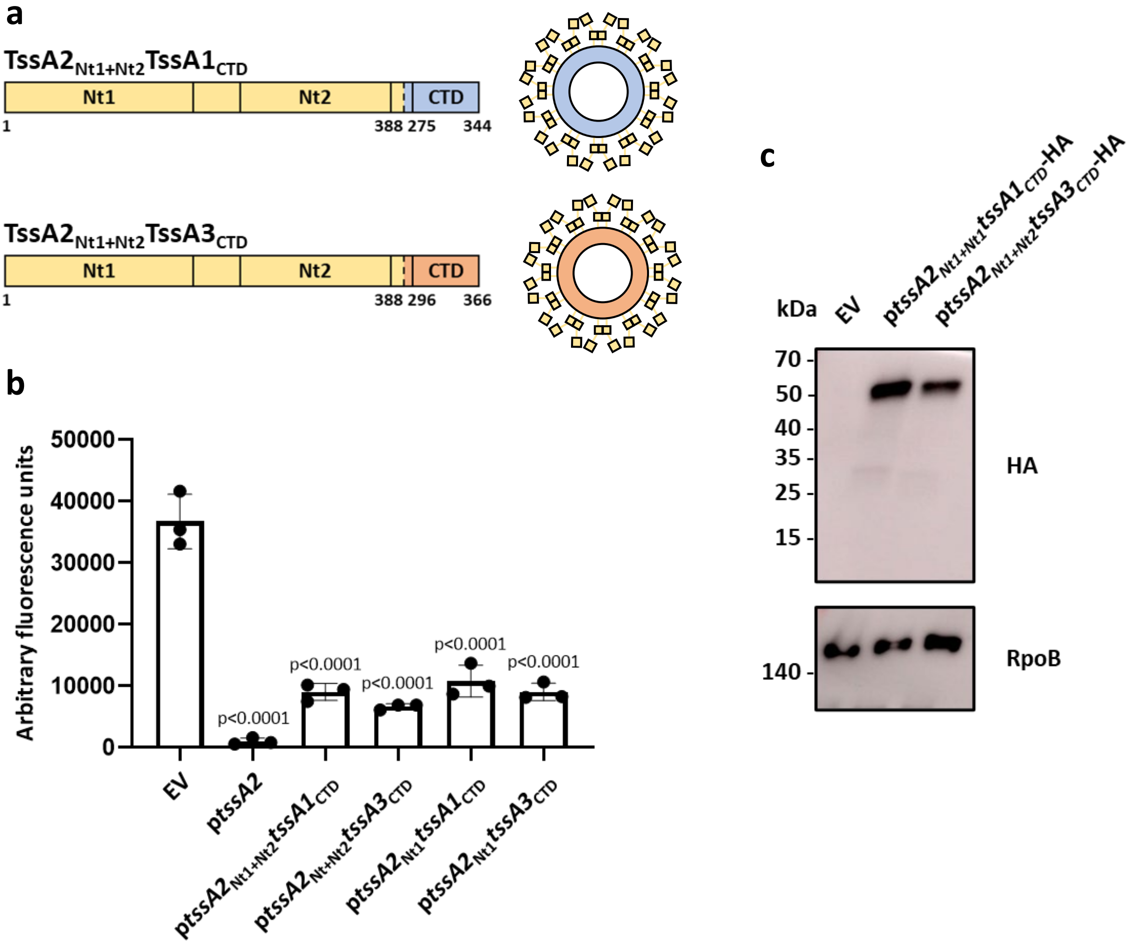


**Supplementary Fig. 9** Assessment of functionality of chimeras with TssA2 N-terminal regions. **a** Schematic of chimeric TssA proteins with TssA2 Nt1 and Nt2 domains, with domain positions and predicted structural organisation **b** To assess complementation of *tssA2* mutant by TssA2 N-terminal domain-containing chimeras, pBBR1MCS4 was introduced either as empty vector (EV), encoding wild type or chimeric TssA2 proteins containing TssA2 N-terminal domains (either Nt1 domain alone or with the Nt2 domain) linked to TssA1 and TssA3 C-terminal domains. H2-T6SS competition assay to assess the T6SS-dependent killing of a GFP-encoding *E. coli* prey by *P. aeruginosa* attacker strains in the PAO1Δ*rsmA*Δ*tssA2* background. (n=3 independent experiments). Statistical testing was conducted by one-way ANOVA with Dunnett's multiple comparisons test, each strain was compared to the parental EV strain. Values are presented as means, error bars represent standard deviation. **c** Expression of chimeric TssA2 Nt1+Nt2 domain-containing proteins. To detect their production, chimeric TssA proteins were C-terminally tagged with hemagglutinin (HA) and expressed from the pBBR1MCS4 vector in the PAO1Δ*rsmA* background. Western blot analysis was carried out of HA-tagged chimeric proteins, with RpoB used as a loading control.

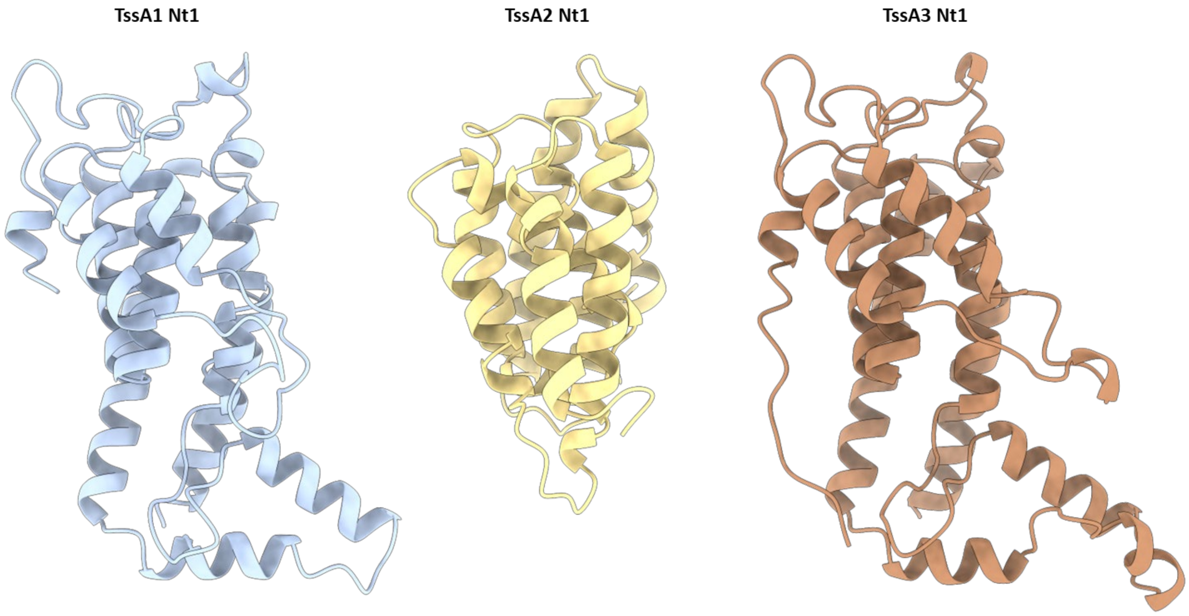


**Supplementary Fig. 10** Homology molecular models of *P. aeruginosa* TssA Nt1 domains. Structural models of the *P. aeruginosa* TssA1 (blue), TssA2 (yellow) and TssA3 (orange) Nt1 domains. The Nt1 domains of the short TssA1 and TssA3 have an extension that is not present in the long TssA2.

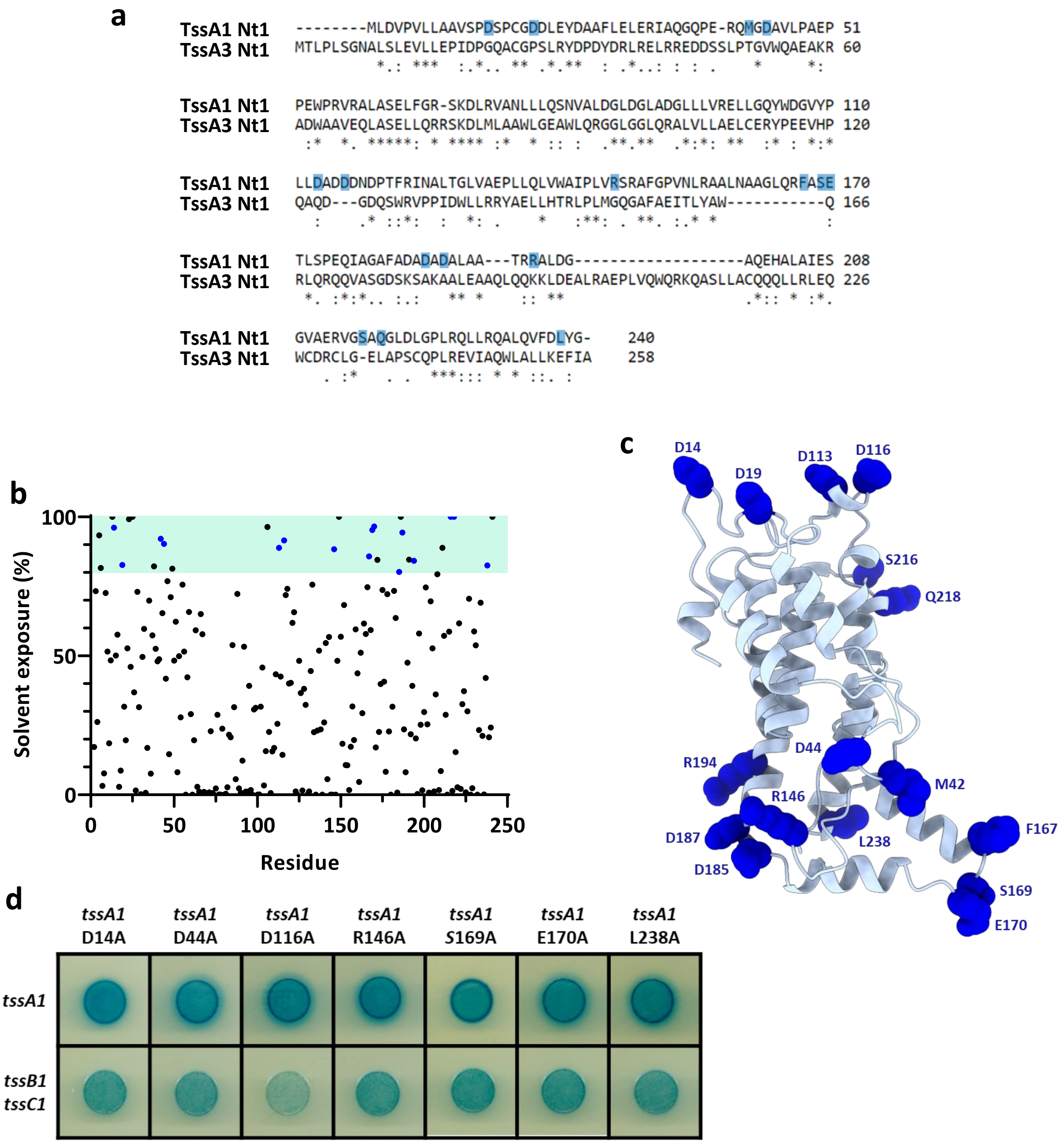


**Supplementary Fig. 11** Identification and screening of candidate TssA1 Nt1 domain residues for sheath interaction. **a** Pairwise sequence alignment between the Nt1 domains of TssA1 and TssA3, generated using Clustal Omega. Non-conserved, surface exposed residues highlighted in blue. **b** Predicted solvent accessibility of TssA1 Nt1 domain residues based on the structural model using GetArea. A solvent exposure value over 80 % (light blue box) was considered surface exposed. Non-conserved, surface exposed residues highlighted in blue. **c** Position of non-conserved, surface exposed residues (blue) in a predicted model of the TssA1 Nt1 domain structure. **d** BTH interactions of TssA1 point mutants with TssA1 and H1-T6SS sheath components.

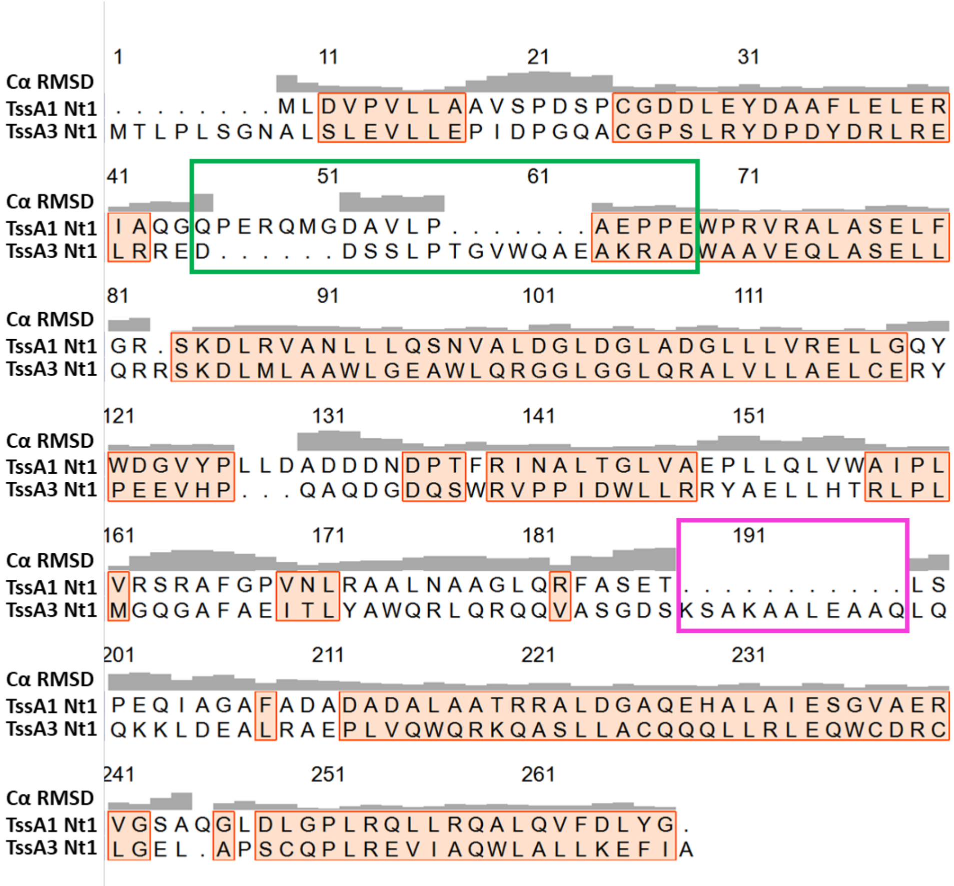

**Supplementary Fig. 12** Alignment of TssA1 and TssA3 Nt1 domain sequences. Sequence alignment where bars positioned above each residue represent the RMSD for the corresponding Cα atoms. Tall bars indicate that Cα atoms are far apart in both structures, while residues within the orange box have an RMSD value close to 1. The sequences corresponding to loops and hairpins are highlighted within empty green and pink rectangles, respectively.

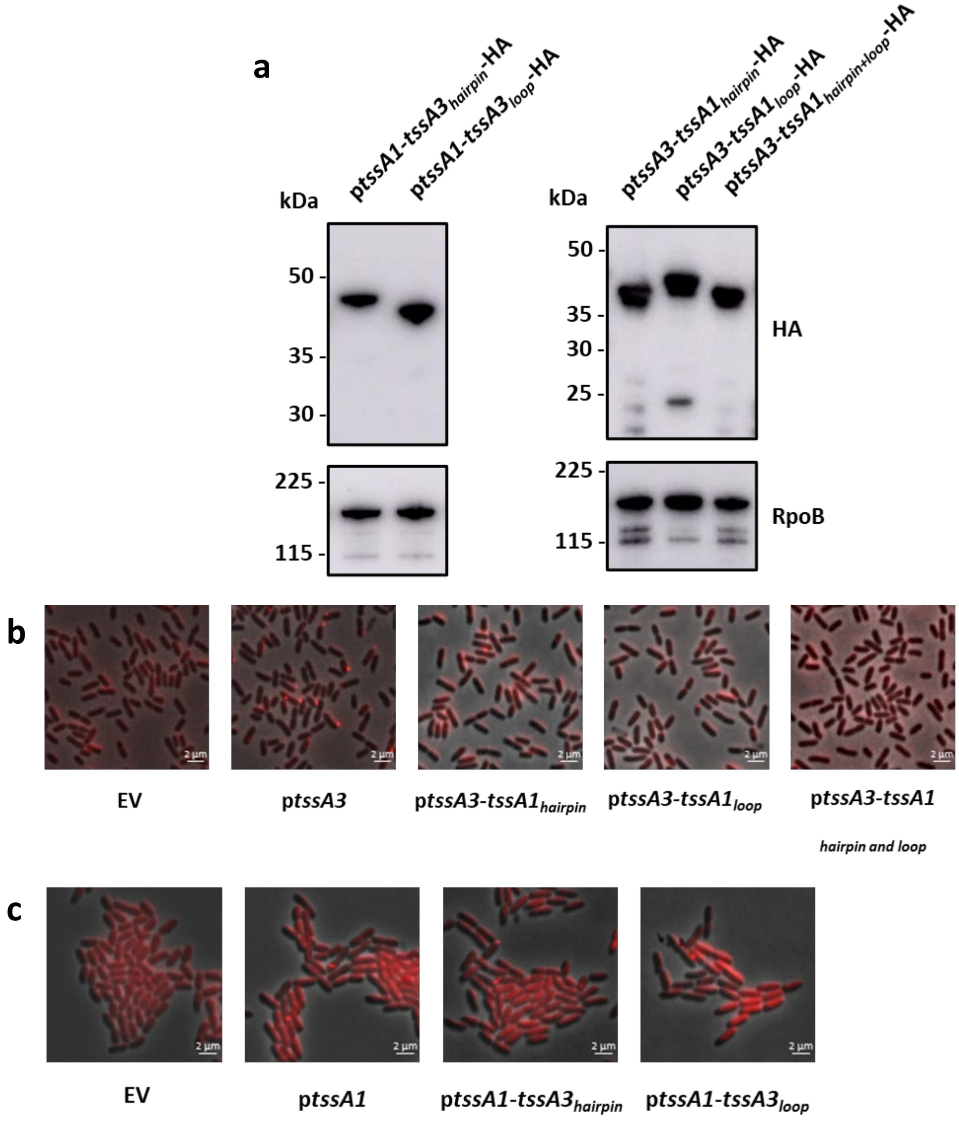


**Supplementary Fig. 13** Assessment of expression and functionality of TssAs proteins with exchanged hairpin an/or loop regions. **a** Expression of TssAs proteins with exchanged hairpin and/or loop regions. To detect their production, TssA proteins were tagged at the C-terminal end with hemagglutinin (HA) and expressed from the pBBR1MCS4 vector in *P. aeruginosa*. Production of TssA1 proteins containing TssA3 hairpin or loop regions was assessed in the PAO1*ΔretS* background, while production of TssA3 proteins containing TssA1 hairpin and/or loop regions was assessed in the PAO1Δ*rsmA* background. Western blot analysis was carried out of HA-tagged chimeric proteins, with RpoB used as a loading control. Production of *tssA1-tssA3*$_{hairpin\ +loop}$ was not stable, so was not included in functional assays. **b** Representative images of TssB3-mScarlet sheath formation in the PAO1Δ*rsmA*Δ*tssA3 tssB3-mScarlet* background, with either pBBR1MCS4 empty vector (EV), wildtype *tssA3* or *tssA3* with exchanged hairpin or loop regions. Scale bar represents 2 µm. **c** Representative images of

TssB1-mScarlet sheath formation in the PAO1Δ*retS*Δ*tssA1 tssB1-mScarlet* background, with either pBBR1MCS4 empty vector (EV), wildtype *tssA1* or *tssA1* with exchanged hairpin or loop regions.

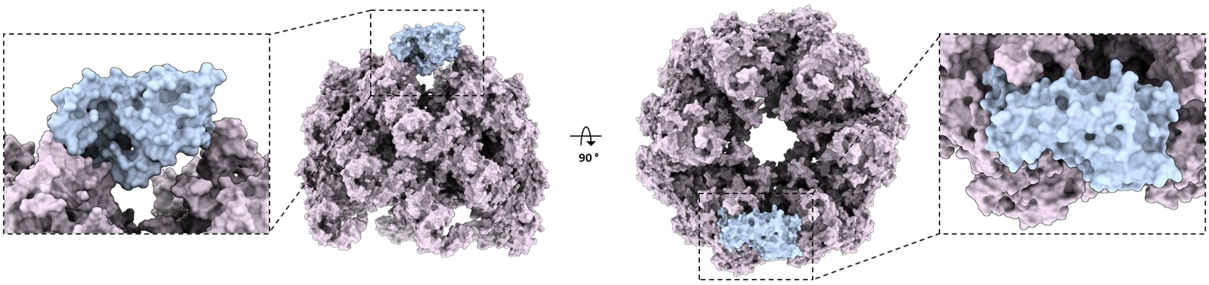


**Supplementary Fig. 14** Model for the docking of the TssA1 Nt1 domain (light blue) at the distal end of the extended sheath (light purple). Side and top views are given.

**Supplementary Table 1.** Primers used in this study.

| Primer Name | Sequence |
|---|---|
| **Vector screening primers** | |
| M13F | TGTAAAACGACGGCCAGT |
| M13R | CAGGAAACAGCTATGACC |
| T25F | GTGACCAGCGGCGATTCGGTGACCGATTAC |
| T25R | TGGCGAAAGGGGGATGTGCTGCAAGGCGAT |
| T18CF | GAAGTTCTCGCCGGATGTACTGGAAACGGT |
| T18CR | TTGGCGGGTGTCGGGGCTGGCTTAACTATG |
| Rpkn | CATATCACAACGTGCGTGGA |
| Upkn | CCCTGGATTTCACTGATGAG |
| T7 promoter | TAATACGACTCACTATAGGG |
| T7 terminator | GCTAGTTATTGCTCAGCGG |
| DuetUP1 | GGATCTCGACGCTCTCCCT |
| DuetDOWN1 | GATTATGCGGCCGTGTACAA |
| ***P. aeruginosa*** **mutator construction primers** | |
| ΔtssA1 P1 | ATATTTCTAGAGTTGGTGCTGGTGTCGGTGAGGTAGTAC |
| ΔtssA1 P2 | TTACTCGCTATCCAGCACGGTGACGATCTCCC |
| ΔtssA1 P3 | GTGCTGGAAAGCGAGTAACGCCTAGCGG |
| ΔtssA1 P4 | AATAAGGATCCTTGCCGTCCATGTAGGTCAGCAGG |
| ΔtssA2 P1 | TGCCTTCTAGAAACAAGTGAATTGAGCAAGAAGTTGCGCA |
| ΔtssA2 P2 | CTATTCGAGATAGGTCATCCTAACCCTTCAATGCACACC |
| ΔtssA2 P3 | ATGACCTATCTCGAATAGGGCTTCGCGCCC |
| ΔtssA2 P4 | ATATAGGATCCCCCAGCTCGCCCAGCACACG |
| ΔtssA3 P1 | ATATGTCTAGAAGCCGCACCACCTCGCGTTCCTGC |
| ΔtssA3 P2 | ATGACCCTGCTGCCCTAGGAGGCGTGGCTATATCTTT |
| ΔtssA3 P3 | CTAGGGCAGCAGGGTCATGGGGTACCTCCGGTGC |
| ΔtssA3 P4 | TATTAGGATCCCTGCTCGGCGTCGCCCTGGACATCG |
| TssB1-mScarlet P1 | TAATAAGGATCCAGGGAGAAACAAGATGGGAAGCA |
| TssB1-mScarlet P2 | TGCTGCTGCCGCCTGCGGCTCGTCGTCTTT |
| TssB1-mScarlet P3 | CCGCAGGCGGCAGCAGCAGGAGGAGGA |
| TssB1-mScarlet P4 | GGAATCCTCTTACGAACCCTTATACAGTTCGTCCAT |
| TssB1-mScarlet P5 | GGTTCGTAAGAGGATTCCAGCATGGCCGA |
| TssB1-mScarlet P6 | TAATAAGGGCCCGCCGAACTGGCCGTATTCCTCTTC |
| TssA2 truncation P3 | GAGACCGTGTAGGGCTTCGCGCCCTCAATTCTC |
| TssB2-mScarlet-I P2 | TGCTGCTGCGGCGTCCTGGGAGGGGGCGGC |
| TssB2-mScarlet-I P3 | CAGGACGCCGCAGCAGCAGGAGGAGGA |
| TssB2-mScarlet-I P4 | GGGGGTGGCTCACGAACCCTTATACAGTTCGTCCATGCCGC |
| TssB2-mScarlet-I P5 | GGTTCGTGAGCCACCCCCTAGCCAAGGAAG |
| TssB2-mScarlet-I P6 | TAATTCGATGATGGCGCCGACCGGCTG |
| TssB3-mScarlet-I P1 | ATATTCATTGCATCGGAGTCAGCCGCCATGGCCGAG |
| TssB3-mScarlet-I P2 | TGCTGCTGCGGCCGGCTGGTCGGCCGGGGG |
| TssB3-mScarlet-I P3 | CAGCCGGCCGCAGCAGCAGGAGGAGGA |
| TssB3-mScarlet-I P4 | GAAGAGGGTCACGAACCCTTATACAGTTCGTCCATGCCGCC |
| TssB3-mScarlet-I P5 | GGTTCGTGACCCTCTTCCCGGAGAAGCCG |
| TssB3-mScarlet-I P6 | TATATAGGCCGATGTCCTGCGGATGGCGACCG |
| ΔrsmN P1 | ATGATGGGCCCGCTCCAGGTTGAGCTGATTGAGGC |

| | |
|---|---|
| ΔrsmN P2 | CAACTCGTCGAAACCCATGTTCCGCGT |
| ΔrsmN P3 | ATGGGTTTCGACGAGTTGAAGACGGCACCG |
| ΔrsmN P4 | ATCATGGATCCTAATCGCGTTCGGCCTGCTG |
| ***P. aeruginosa* mutant screening primers** | |
| TssA1 exF | AAGTCGAACAGGTCGGGCTTGGTGT |
| TssA1 exR | ATCGACTTGATCGCGTCGTTGGAGAT |
| TssA2 exF | TCTCTGTCGTCGCCGTACGC |
| TssA2 exR | AGCGCCTGGAAATCGGGATG |
| TssA3 exF | GGCTCGCGCTGGTGTAGC |
| TssA3 exR | CCAGGACAGTGCCGAGCTG |
| TssB1 exF | CGCCAACCGCGAAGACGTCCT |
| TssB1 exR | CTTCCTTCAGGGTGATGATCAGGTACT |
| TssB2 exF | CAGGCGATGCGGGAAGTCGAAA |
| TssB2 exR | TCTGCCACTTGGCGAACTGC |
| TssB3 exF | TCCGTGCTTCATGGGAGTCC |
| TssB3 exR | TCGAACAGGCGTGGGCTG |
| RsmN exF | CGGCCCAGTGACAGAGATCG |
| RsmN exR | TGATGGTCCGGCCTTTGTGC |
| RetS exF | GAGGAGGCCAGCTTCATCGTCATG |
| RetS exR | TGCTGCACGTTGTCGCTCTG |
| **Bacterial two-hybrid vector primers** | |
| BTH TssB1 F | GCTCTAGAGATGGGAAGCACTACCAGCAGTCAG |
| BTH TssC1 R | GGGGTACCGCTGTCGTTGAATGCCTCAGGCCTC |
| BTH TssA2 F | TAAATTCTAGAGATGACCTATTCGAGCAAGCTCTCC |
| BTH TssA2 R | TATAAGGATCCCCCTATTCGAGCACCACCTCG |
| BTH TssA3 F | ATTTATCTAGAGATGACCCTGCCTCTTTCCGG |
| BTH TssA3 R | TATAAGGATCCTCCTAGGGCAGTACGCC |
| PA0082BTHFW | GCTCTAGAGGTGCTGGATGTACCCGTT |
| A1 with A3 hairpin R | GGGACTCTGAAGTTGCGCCGCTTCCAGCGCCGCCTTGGCGCTCTTGCTG TCGCCGCTGGCGCGTTGCAGCCCGGCGGCGTTC |
| A1 with A3 hairpin F 2 | GAAGCGGCGCAACTTCAGAGTCCCGAGCAGATCGCCGGCG |
| PA0082BTHRV | GGGGTACCTTACTCGCTTTCCGGGCC |
| PA0082BTHFW | GCTCTAGAGGTGCTGGATGTACCCGTT |
| A1 with A3 loop R | CGGCCAGTCGGCGCGCTTGGCCTCCGCTTGCCAGACGCCGGTCGGCAGG CTGCTGTCGCCCTGGGCGATGCGTTCGAGTTCGAG |
| A1 with A3 loop F | AAGCGCGCCGACTGGCCGCGCGTACGCGCGCTG |
| PA0082BTHRV | GGGGTACCTTACTCGCTTTCCGGGCC |
| BTH TssA3 F | ATTTATCTAGAGATGACCCTGCCTCTTTCCGG |
| A3 with A1 loop R | GCAGCACGGCATCGCCCATCTGGCGCTCGGGTTGGTCCTCCCGGCGCAA CTCGCG |
| A3 with A1 loop F | CAGATGGGCGATGCCGTGCTGCCCGCCGAGCCGCCGGAGTGGGCCGCG GTGGAGCAGTTG |
| BTH TssA3 R | TATAAGGATCCTCCTAGGGCAGTACGCC |
| BTH TssA3 F | ATTTATCTAGAGATGACCCTGCCTCTTTCCGG |
| A3 with A1 hairpin R | CAGGGTCTCGCTGGCGAAGACCTGCTGGCGCTGCAACCTCTG |
| A3 with A1 hairpin F | CAGGTCTTCGCCAGCGAGACCCTGCAGAAGAAGCTAGACGAAGCGTTG CGCG |
| BTH TssA3 R | TATAAGGATCCTCCTAGGGCAGTACGCC |

| | |
|---|---|
| PKT25B2FW | GCTCTAGAGATGGCCAAAGAAGGCTCG |
| PKT25C2RV | CGGAATTCTTACTCTTTGTCCAGCTT |
| TssK2 BTH F | AGAGATCTAGAGATGAACGTGCACAAGATCGTCTGG |
| TssK2 BTH R | GAGAGGAATTCGTTCAGTTCCTGATGGCCCAG |
| TssB3 BTH F | ATTATTCTAGACATGGCCGAGAGTACGCAG |
| TssC3 BTH R | ATAATGGATCCCGGACTCACGCCGCTACC |
| TssK3 BTH F | TTATATCTAGATATGAGTGTTCTGCCTGACGCG |
| TssK3 BTH R | ATATAGGATCCTGTCTAGGTGTTGTCGCTG |
| TssA1 Nt1 BTH R | ATATAGGATCCTTAGCCGTACAGGTCGAACAC |
| TssA1 CTD BTH F | TATTATCTAGAGATCGCCAACCGCGAAG |
| TssA2 Nt1 BTH R | TATATGGATCCTTACTGGTTGTCGGCGGCGCG |
| TssA2 Nt2+CTD BTH F | TTAATTCTAGACGCCCACAAGGCCATG |
| TssA3 Nt1 BTH R | TTATAGGATCCTTAGTGCATGGCGATGAACTCCTTG |
| TssA3 CTD BTH F | ATATATCTAGAGCCGGCTGGCGCGCCGAC |
| ***P. aeruginosa* expression vector primers** | |
| pBBR-tssA1 F | TAATAGTCGACAGGTGAGGGCCGCGCAGCGG |
| pBBR-tssA1 R | ATAATGGATCCTTACTCGCTTTCCGGGCCTCGCAG |
| pBBR-tssA2 F | ATTAAGTCGACGTTAAGATATTCATTGGCGCACATGGTG |
| pBBR-tssA2 R | ATATAGGATCCCTATTCGAGCACCACCTCGAGATC |
| pBBR-tssA3 F | ATTATGTCGACCGCGACGCTGTTGCCGGCCAC |
| pBBR-tssA3 R | TTAATGGATCCCTAGGGCAGTACGCCAAGCAGCGA |
| A1Nt1-A2CTD R | GGTCTCGACGGCTCGCGGTGCCGGCGC |
| A1Nt1-A2CTD F | CCGCGAGCCGTCGAGACCGTGGCCCTG |
| A1Nt1-A2Nt2+CTD R 2 | CATGGCCTTGTGGGCGTCGGCTCGCGGTGCCGGCGC |
| A1Nt1-A2Nt2+CTD F 2 | GCGCCGGCACCGCGAGCCGACGCCCACAAGGCCATG |
| A1Nt1-A3CTD R | GCCAGCCGGGGCTCGCGGTGCCGGCGC |
| A1Nt1-A3CTD F | CCGCGAGCCCCGGCTGGCGCGCCGACC |
| A2Nt1-A1CTD R | CAAGGACTCTTCGGGCTGGTTGTCGGC |
| A2Nt1-A1CTD F | CAGCCCGAAGAGTCCTTGGCGCCGGGTGCC |
| A2Nt1-A3CTD R | GCCAGCCGGGTCGCGCTCGTTGTCGAT |
| A2Nt1-A3CTD F | GAGCGCGACCCGGCTGGCGCGCCGACC |
| A3Nt1-A1CTD R 2 | GCTGGCTCGCCCGCTCGGCGCGCTCG |
| A3Nt1-A1CTD F 2 | GCCGAGCGGGCGAGCCAGCGGCGAGATCGCC |
| A3Nt1-A2CTD R | GGGCATGACTTGCGGGTGCATGGCGAT |
| A3Nt1-A2CTD F | CACCCGCAAGTCATGCCCCATCTCCAG |
| A3Nt1-A2Nt2+CTD R | GCCAGGTTCTTGCGGGTGCATGGCGAT |
| A3Nt1 -A2Nt2+CTD F | CACCCGCAAGAACCTGGCGTGGTCGGC |
| A1 with A3 hairpin R | GGGACTCTGAAGTTGCGCCGCTTCCAGCGCCGCCTTGGCGCTCTTGCTG TCGCCGCTGGCGCGTTGCAGCCCGGCGGCGTTC |
| A1 with A3 hairpin F 2 | GAAGCGGCGCAACTTCAGAGTCCCGAGCAGATCGCCGGCG |
| A1 with A3 loop R | CGGCCAGTCGGCGCGCTTGGCCTCCGCTTGCCAGACGCCGGTCGGCAGG CTGCTGTCGCCCTGGGCGATGCGTTCGAGTTCGAG |
| A1 with A3 loop F | AAGCGCGCCGACTGGCCGCGCGTACGCGCGCTG |
| A3 with A1 hairpin R | CAGGGTCTCGCTGGCGAAGACCTGCTGGCGCTGCAACCTCTG |

| | |
|---|---|
| A3 with A1 hairpin F | CAGGTCTTCGCCAGCGAGACCCTGCAGAAGAAGCTAGACGAAGCGTTG CGCG |
| A3 with A1 loop R | GCAGCACGGCATCGCCCATCTGGCGCTCGGGTTGGTCCTCCCGGCGCAA CTCGCG |
| A3 with A1 loop F | CAGATGGGCGATGCCGTGCTGCCCGCCGAGCCGCCGGAGTGGGCCGCG GTGGAGCAGTTG |
| pBBR4-A1PP F | ATTATGTCGACCGGGTTTTCCGGCGCGC |
| pBBR4-A1PP R | ATAATGGATCCCTAGCGGGTGCCCAGCAC |
| pBBR4-A1PA-A1PP F | ATAATGGATCCCTAGCGGGTGCCCAGCAC |
| pBBR4-A1PA-A1PP R | GGTGCGCAGGGCTCGCGGTGCCGGCG |
| pBBR4-A1PP-A1PA F | CACCCCGCCGAGTCCTTGGCGCCGGGTGC |
| pBBR4-A1PP-A1PA R | CAAGGACTCGGCGGGGTGTAGCTCTACGCC |
| pBBR4-A2NN-A1C F | CGCAAGGTCGCCAGCGGCGAGATCGCC |
| pBBR4-A2NN-A1C R | GCCGCTGGCGACCTTGCGCGGCGCACTG |
| pBBR4-A2NN-A3C F | CGCAAGGTCCCGGCTGGCGCGCCGAC |
| pBBR4-A2NN-A3C R | GCCAGCCGGGACCTTGCGCGGCGCACTG |
| ***E. coli* expression vector primers** | |
| TssA1 CTD F | ATATTGAATTCAGCCAGCGGCGAGATC |
| Duet-TssA1 R | TGGCCAAGCTTCCGCTAGGCGTTACTCG |
| Duet TssA2 CTD F | TAATTGAATTCGCGCAAGGTCGAGACCGT |
| Duet-TssA2 R | ATATTAAGCTTGCGCGAAGCCCTATTCGAG |
| TssA3 CTD F | ATTTAGAATTCGCCGGCTGGCGCGCCGA |
| Duet-TssA3 R | ATTTTAAGCTTGCCACGCCTCCTAGGGC |
| **Site-directed mutagenesis primers** | |
| TssA1 D14A F | GCTGCCGTATCCCCGGCTTCGCCCTGTGGCGACG |
| TssA1 D14A R | CGTCGCCACAGGGCGAAGCCGGGGATACGGCAGC |
| TssA1 D44A F | CGCCAGATGGGCGCTGCCGTGCTGCCCGCC |
| TssA1 D44A R | GGCGGGCAGCACGGCAGCGCCCATCTGGCG |
| TssA1 D116A F | CTATCCGTTGCTCGACGCCGATGCTGACAACGACCCCACCTTCCG |
| TssA1 D116A R | CGGAAGGTGGGGTCGTTGTCAGCATCGGCGTCGAGCAACGGATAG |
| TssA1 R146A F | GATCCCGCTGGTGGCTTCGCGGGCGTTCGG |
| TssA1 R146A R | CCGAACGCCCGCGAAGCCACCAGCGGGATC |
| TssA1 S169A F | GGCTGCAACGCTTCGCCGCTGAGACCCTGAGTCCCG |
| TssA1 S169A R | CGGGACTCAGGGTCTCAGCGGCGAAGCGTTGCAGCC |
| TssA1 E170A F | CTGCAACGCTTCGCCAGCGCTACCCTGAGTCCCGAGCAG |
| TssA1 E170A R | CTGCTCGGGACTCAGGGTAGCGCTGGCGAAGCGTTGCAG |
| TssA1 L238A F | CCTCCAGGTGTTCGACGCTTACGGCCCGCAGGGCG |
| TssA1 L238A R | CGCCCTGCGGGCCGTAAGCGTCGAACACCTGGAGG |
| pBBR-A1PP-HA_F | GTATGGGTAACCACTACCGCGGGTGCCCAGCA |
| pBBR-A1PP-HA_R | GATGTTCCAGATTACGCTTAGGGATCCACTAGTTCTAGAGCG |
| pBBR-A1-HA_F | GTATGGGTAACCACTACCCTCGCTTTCCGGGC |
| pBBR-A1-HA_R | GATGTTCCAGATTACGCTTAAGGATCCACTAGTTCTAGAG |
| pBBR-A2-HA_F | GTATGGGTAACCACTACCTTCGAGCACCACCTC |
| pBBR-A3-HA_F | GTATGGGTAACCACTACCGGGCAGTACGCCAAG |
| pBBR-A2/A3-HA_R | GATGTTCCAGATTACGCTTAGGGATCCACTAGTTCTAG |
| HA Primer F | GCGTAATCTGGAACATCG |

**Supplementary Table 2.** Strains used in this study.

| Strain | Description | Source |
|---|---|---|
| | ***E. coli*** | |
| DH5α omnimax | F′ {*pro*AB *lac*Iq *lac*Z ΔM15 *Tn*10 (TetR) Δ(*ccdA*B)} *mcr*A Δ(*mrr hsd*RMS-*mcr*BC) Φ 80(*lac*Z)ΔM15 Δ(*lac*ZYA-*arg*F)U169 *end*A1 *rec*A1 *sup*E44 *thi*-1 *gyr*A96 *rel*A1 *ton*A *pan*D | Invitrogen |
| DHM1 | Strain used for bacterial two-hybrid, $F^-$ , *cya-854, recA1, endA1, gyrA96* ($Nal^R$), *thi1, hsdR17, spoT1, rfbD1, glnV44(AS).* | (Karimova *et al.*, 1998)[1] |
| CC118λ*pir* | Host strain for pKNG101, Δ(*ara-leu*) *araDΔlacX74 galE galK-phoA20 thi-1 rpsE rpoB argE*($Amp^R$) *recA1 Rfrλpir* | (Herrero, de Lorenzo and Timmis, 1990)[2] |
| BL21 (λDE3) | Strain used for protein expression, $F^–$ *dcm lon ompT hsdS*($r_B$ – $m_B$ – ) *gal* λ(DE3 [*lacI lacUV5-T7p07 ind1 sam7 nin5*]) | Stratagene |
| 1047 | Helper strain for three-partner conjugation, ($Km^R$), *oriColE1 RK2*$^-$*Mob*$^+$*RK2*$^-$*Tra*$^+$. | (Figurski and Helinski, 1979)[3] |
| | ***P. aeruginosa*** | |
| PAO1Δ*rsmAΔrsmN* | PAO1 with deletion in *rsmA* (PA0905), *rsmN* (PA5183.1). | This study. |
| PAO1Δ*rsmAΔrsmN*Δ*tssB1* | PAO1 with deletion in *rsmA* (PA0905), *rsmN* (PA5183.1) and *tssB1* (PA0083). | This study. |
| PAO1Δ*rsmAΔrsmN*Δ*tssA1* | PAO1 with deletion in *rsmA* (PA0905), *rsmN* (PA5183.1) and *tssA1* (PA0082). | This study. |
| PAO1Δ*rsmA* | PAO1 with deletion in *rsmA* (PA0905). | (Allsopp *et al.*, 2017)[4] |
| PAO1Δ*rsmAΔtssB2* | PAO1 with deletion in *rsmA* (PA0905) and *tssB2* (PA1657). | This study. |
| PAO1Δ*rsmAΔtssA2* | PAO1 with deletion in *rsmA* (PA0905) and *tssA2* (PA1656). | This study. |
| PAO1Δ*retS* | PAO1 with deletion in *retS* (PA4856). | (Rudzite 2023)[5] |
| PAO1Δ*retS*Δ*tssA1 tssB1-mScarlet-I* | PAO1 with deletion in *retS* (PA4856) and *tssA1* (PA0082), with chromosomal fusion of mScarlet-I at the C-terminus of *tssB1* (PA0083). | This study. |
| PAO1Δ*rsmA*Δ*tssA2 tssB2-mScarlet-I* | PAO1 with deletion in *rsmA* (PA0905) and *tssA2* (PA1656) with chromosomal fusion of mScarlet-I at the C-terminus of *tssB2* (PA1657). | This study. |
| PAO1Δ*rsmA*Δ*tssA3 tssB3-mScarlet-I* | PAO1 with deletion in *rsmA* (PA0905) and *tssA3* (PA2360) with chromosomal fusion of mScarlet-I at the C-terminus of *tssB3* (PA2365). | This study. |

**Supplementary Table 3.** Plasmids used in this study.

| Plasmid | Description | Source |
|---|---|---|
| | **Bacterial two-hybrid vectors** | |
| pKT25 | Bacterial two-hybrid vector, encoding the T25 fragment of *B. pertussis cyaA,* with the MCS at the C-terminal end of T25, $Km^R$. | (Karimova, Ullmann and Ladant, 2001)[6] |
| pUT18C | Bacterial two-hybrid vector, encoding T18 fragment of *B. pertussis cyaA*, with the MCS at the C-terminal end of T18, $Amp^R$. | (Karimova, Ullmann and Ladant, 2001)[6] |
| pKT25-*tssA1* | pKT25 encoding *tssA1* (PA0082), $Km^R$. | (Lossi *et al*., 2012)[7] |
| pKT25-*tssA1* D14A | pKT25 encoding *tssA1* (PA0082) with D14A substitution, $Km^R$. | This study. |
| pKT25-*tssA1* Q41A | pKT25 encoding *tssA1* (PA0082) with Q41A substitution, $Km^R$. | This study. |
| pKT25-*tssA1* D44A | pKT25 encoding *tssA1* (PA0082) with D44A substitution, $Km^R$. | This study. |
| pKT25-*tssA1* D116A | pKT25 encoding *tssA1* (PA0082) with D116A substitution, $Km^R$. | This study. |
| pKT25-*tssA1* R146A | pKT25 encoding *tssA1* (PA0082) with R146A substitution, $Km^R$. | This study. |
| pKT25-*tssA1* R148A | pKT25 encoding *tssA1* (PA0082) with R148A substitution, $Km^R$. | This study. |
| pKT25-*tssA1* S169A | pKT25 encoding *tssA1* (PA0082) with S169A substitution, $Km^R$. | This study. |
| pKT25-*tssA1* E170A | pKT25 encoding *tssA1* (PA0082) with E170A substitution, $Km^R$. | This study. |
| pKT25-*tssA1* L238A | pKT25 encoding *tssA1* (PA0082) with L238A substitution, $Km^R$. | This study. |
| pKT25-$tssA1_{Nt1}$ | pKT25 encoding *tssA1* (PA0082) Nt1 domain residues 1-240, $Km^R$. | This study. |
| pKT25-$tssA1_{CTD}$ | pKT25 encoding *tssA1* (PA0082) CTD residues 275-344, $Km^R$. | This study. |
| pKT25-*tssA2* | pKT25 encoding *tssA2* (PA1656), $Km^R$. | This study. |
| pKT25-$tssA2_{Nt1}$ | pKT25 encoding *tssA2* (PA2360) Nt1 domain residues 1- 190, $Km^R$. | This study. |
| pKT25-$tssA2_{Nt2+CTD}$ | pKT25 encoding *tssA2* (PA1656) Nt2+CTD residues 222-528, $Km^R$. | This study. |
| pKT25-*tssA3* | pKT25 encoding *tssA3* (PA2360), $Km^R$. | This study. |
| pKT25-$tssA3_{Nt1}$ | pKT25 encoding *tssA3* (PA2360) Nt1 domain residues 1- 260, $Km^R$. | This study. |
| pKT25-$tssA3_{CTD}$ | pKT25 encoding *tssA3* (PA2360) CTD residues 296-366, $Km^R$. | This study. |

| | | |
|---|---|---|
| pKT25-*tssA1*$_{Nt1}$-*tssA2*$_{CTD}$ | pKT25 encoding *tssA1*$_{Nt1}$ (PA0082) (1-275) *tssA2*$_{CTD}$ (PA1656) (388-518) chimera, Km$^R$. | This study. |
| pKT25-*tssA1*$_{Nt1}$-*tssA2*$_{Nt2+CTD}$ | pKT25 encoding *tssA1*$_{Nt1}$ (PA0082) (1-275) *tssA2*$_{Nt2+CTD}$ (PA1656) (222-518) chimera, Km$^R$. | This study. |
| pKT25-*tssA1*$_{Nt1}$-*tssA3*$_{CTD}$ | pKT25 encoding *tssA1*$_{Nt1}$ (PA0082) (1-275) *tssA3*$_{CTD}$ (PA2360) (296-366) chimera, Km$^R$. | This study. |
| pKT25-*tssA2*$_{Nt1}$-*tssA1*$_{CTD}$ | pKT25 encoding *tssA2*$_{Nt1}$ (PA1656) (1-192) *tssA1*$_{CTD}$ (PA0082) (246-344) chimera, Km$^R$. | This study. |
| pKT25-*tssA2*$_{Nt1}$-*tssA3*$_{CTD}$ | pKT25 encoding *tssA2*$_{Nt1}$ (PA1656) (1-222) *tssA3*$_{CTD}$ (PA2360) (296-366) chimera, Km$^R$. | This study. |
| pKT25-*tssA3*$_{Nt1}$-*tssA1*$_{CTD}$ | pKT25 encoding *tssA3*$_{Nt1}$ (PA2360) (1-262) *tssA1*$_{CTD}$ (PA0082) (246-344) chimera, Km$^R$. | This study. |
| pKT25-*tssA3*$_{Nt1}$-*tssA2*$_{CTD}$ | pKT25 encoding *tssA3*$_{Nt1}$ (PA2360) (1-262) *tssA2*$_{CTD}$ (PA1656) (375-518) chimera, Km$^R$. | This study. |
| pKT25-*tssA3*$_{Nt1}$-*tssA2*$_{Nt2+CTD}$ | pKT25 encoding *tssA3*$_{Nt1}$ (PA2360) (1-262) *tssA2*$_{Nt2+CTD}$ (PA1656) (192-518) chimera, Km$^R$. | This study. |
| pKT25-*tssA1*-*tssA3*$_{loop}$ | pKT25 encoding *tssA1* (PA0082) with loop region (Q37-E53) replaced with *tssA3* (PA2360) loop region (D46-D62), Km$^R$. | This study. |
| pKT25-*tssA1*-*tssA3*$_{hairpin}$ | pKT25 encoding *tssA1* (PA0082) with hairpin region (F169-L172) replaced with *tssA3* (PA2360) hairpin region (A174-Q191), Km$^R$. | This study. |
| pKT25-*tssA3*-*tssA1*$_{loop}$ | pKT25 encoding *tssA3* (PA2360) with loop region (D46-D62) replaced with *tssA1* (PA0082) loop region (Q37-E53), Km$^R$. | This study. |
| pKT25-*tssA3*-*tssA1*$_{hairpin}$ | pKT25 encoding *tssA3* (PA2360) with hairpin region (A174-Q191) replaced with *tssA1* (PA0082) hairpin region (F169-L172), Km$^R$. | This study. |
| pKT25-*tssA3*-*tssA1*$_{hairpin\ and\ loop}$ | pKT25 encoding *tssA3* (PA2360) with hairpin (A174-Q191) and loop (D46-D62) regions replaced with *tssA1* (PA0082) hairpin (F169-L172) and loop (Q37-E53) regions, Km$^R$. | This study. |
| pUT18C-*tssA1* | pUT18C encoding *tssA1* (PA0082), Amp$^R$. | (Planamente *et al.*, 2016)[8] |
| pUT18C-*tssA1*$_{Nt1}$ | pUT18C encoding *tssA1* (PA0082) Nt1 domain residues 1-240, Amp$^R$. | This study. |
| pUT18C-*tssA1*$_{CTD}$ | pUT18C encoding *tssA1* (PA0082) CTD residues 275-344, Amp$^R$. | This study. |
| pUT18C-*tssB1* | pUT18C encoding *tssB1* (PA0083), Amp$^R$. | (Lossi *et al.*, 2013)[9] |
| pUT18C-*tssC1* | pUT18C encoding *tssC1* (PA0084), Amp$^R$. | (Lossi *et al.*, 2013)[9] |

| | | |
|---|---|---|
| pUT18C-*tssB1tssC1* | pUT18C encoding *tssB1* and *tssC1* (PA0083-84), $Amp^R$. | This study. |
| pUT18C-*tssA2* | pUT18C encoding *tssA2* (PA1656), $Amp^R$. | This study. |
| pUT18C-$tssA2_{Nt1}$ | pUT18C encoding *tssA2* (PA1656) Nt1 domain residues 1- 190, $Amp^R$. | This study. |
| pUT18C-$tssA2_{Nt2+CTD}$ | pUT18C encoding *tssA2* (PA1656) Nt2+CTD residues 222-528, $Amp^R$. | This study. |
| pUT18C-*tssB2* | pUT18C encoding *tssB2* (PA1657), $Amp^R$. | (Lossi *et al.,* 2013)[9] |
| pUT18C-*tssC2* | pUT18C encoding *tssC2* (PA1658), $Amp^R$. | (Lossi *et al.,* 2013)[9] |
| pUT18C-*tssB2tssC2* | pUT18C encoding *tssB2* and *tssC2* (PA1657-1658), $Amp^R$. | This study. |
| pUT18C-*tssA3* | pUT18C encoding *tssA3* (PA2360), $Amp^R$. | This study. |
| pUT18C-$tssA3_{Nt1}$ | pUT18C encoding *tssA3* (PA2360) Nt1 domain residues 1- 260, $Amp^R$. | This study. |
| pUT18C-$tssA3_{CTD}$ | pUT18C encoding *tssA3* (PA2360) CTD residues 296-366, $Amp^R$. | This study. |
| pUT18C-*tssB3* | pUT18C encoding *tssB3* (PA2365), $Amp^R$. | This study. |
| pUT18C-*tssC3* | pUT18C encoding *tssC3* (PA2366), $Amp^R$. | This study. |
| pUT18C-*tssB3tssC3* | pUT18C encoding *tssB3* and *tssC3* (PA2365-2366), $Amp^R$. | This study. |
| **Expression vectors** | | |
| pBBR1MCS4 | Broad host range vector, with constitutive $P_{LAC}$ promoter, $Amp^R$/$Carb^R$. | (Kovach *et al.*, 1995)[10] |
| pBBR1MCS4-*tssA1* | pBBR1MCS4 encoding *tssA1* (PA0082), $Amp^R$/$Carb^R$. | This study. |
| pBBR1MCS4-*tssA2* | pBBR1MCS4 encoding *tssA2* (PA1656), $Amp^R$/$Carb^R$. | This study. |
| pBBR1MCS4-*tssA3* | pBBR1MCS4 encoding *tssA3* (PA2360), $Amp^R$/$Carb^R$. | This study. |
| pBBR1MCS4-$tssA1_{Nt1}$-$tssA2_{CTD}$ | pBBR1MCS4 encoding *tssA1* Nt1 (1-275) *tssA2* CTD (388-518) chimera, $Amp^R$/$Carb^R$. | This study. |
| pBBR1MCS4-$tssA1_{Nt1}$-$tssA2_{Nt2+CTD}$ | pBBR1MCS4 encoding *tssA1* Nt1 (1-275) *tssA2* Nt2+CTD (222-518) chimera, $Amp^R$/$Carb^R$. | This study. |
| pBBR1MCS4-$tssA1_{Nt1}$-$tssA3_{CTD}$ | pBBR1MCS4 encoding *tssA1* Nt1 (1-275) *tssA3* CTD (296-366) chimera, $Amp^R$/$Carb^R$. | This study. |
| pBBR1MCS4-$tssA2_{Nt1}$-$tssA1_{CTD}$ | pBBR1MCS4 encoding *tssA2* Nt1 (1-192) *tssA1* CTD (246-344) chimera, $Amp^R$/$Carb^R$. | This study. |
| pBBR1MCS4-$tssA2_{Nt1}$-$tssA3_{CTD}$ | pBBR1MCS4 encoding *tssA2* Nt1 (1-222) *tssA3* CTD (296-366) chimera, $Amp^R$/$Carb^R$. | This study. |

| | | |
|---|---|---|
| pBBR1MCS4-$tssA3_{Nt1}$-$tssA1_{CTD}$ | pBBR1MCS4 encoding *tssA3* Nt1 (1-262) *tssA1* CTD (246-344) chimera, $Amp^R/Carb^R$. | This study. |
| pBBR1MCS4-$tssA3_{Nt1}$-$tssA2_{CTD}$ | pBBR1MCS4 encoding *tssA3* Nt1 (1-262) *tssA2* CTD (375-518) chimera, $Amp^R/Carb^R$. | This study. |
| pBBR1MCS4-$tssA3_{Nt1}$-$tssA2_{Nt2+CTD}$ | pBBR1MCS4 encoding *tssA3* Nt1 (1-262) *tssA2* Nt2+CTD (192-518) chimera, $Amp^R/Carb^R$. | This study. |
| pBBR1MCS4-*tssA1*-$tssA3_{hairpin}$ | pBBR1MCS4 encoding *tssA1* with hairpin region (F169-L172) replaced with *tssA3* hairpin region (A174-Q191), $Amp^R/Carb^R$. | This study. |
| pBBR1MCS4-*tssA1*-$tssA3_{loop}$ | pBBR1MCS4 encoding *tssA1* with loop region (Q37-E53) replaced with *tssA3* loop region (D46-D62), $Amp^R/Carb^R$. | This study. |
| pBBR1MCS4-*tssA3*-$tssA1_{hairpin}$ | pBBR1MCS4 encoding *tssA3* with hairpin region (A174-Q191) replaced with *tssA1* hairpin region (F169-L172), $Amp^R/Carb^R$. | This study. |
| pBBR1MCS4-*tssA3*-$tssA1_{loop}$ | pBBR1MCS4 encoding *tssA3* with loop region (D46-D62) replaced with *tssA1* loop region (Q37-E53), $Amp^R/Carb^R$. | This study. |
| pBBR1MCS4-*tssA3*-$tssA1_{hairpin\ and\ loop}$ | pBBR1MCS4 encoding *tssA3* with hairpin (A174-Q191) and loop (D46-D62) regions replaced with *tssA1* hairpin (F169-L172) and loop (Q37-E53) regions, $Amp^R/Carb^R$. | This study. |
| pBBR1MCS4-$tssA2_{Nt1+Nt2}$-$tssA1_{CTD}$ | pBBR1MCS4 encoding $tssA2_{Nt1+Nt2}$ (1-388) and $tssA1_{CTD}$ (275-344) chimera, $Amp^R/Carb^R$ | This study. |
| pBBR1MCS4-$tssA2_{Nt1+Nt2}$-$tssA3_{CTD}$ | pBBR1MCS4 encoding $tssA2_{Nt1+Nt2}$ (1-388) and $tssA3_{CTD}$ (296-366) chimera, $Amp^R/Carb^R$ | This study. |
| pBBR1MCS4-$tssA1^{PP}$ | pBBR1MCS4 encoding full length *P. putida tssA1* (PP3088), $Amp^R/Carb^R$ | This study. |
| pBBR1MCS4-$tssA1^{PA}{}_{Nt1}$-$tssA1^{PP}{}_{CTD}$ | pBBR1MCS4 encoding $tssA1^{PA}{}_{Nt1}$ (1-275) and $tssA1^{PP}{}_{CTD}$ (22-361) chimera. $Amp^R/Carb^R$ | This study. |
| pBBR1MCS4-$tssA1^{PP}{}_{Nt1}$-$tssA1^{PA}{}_{CTD}$ | pBBR1MCS4 encoding $tssA1^{PP}{}_{Nt1}$ (1-259) and $tssA1^{PA}{}_{CTD}$ (246-344) chimera, $Amp^R/Carb^R$ | This study. |
| pBBR1MCS4-*tssA1-HA* | pBBR1MCS4 encoding C-terminal HA tagged *tssA1* (PA0082), $Amp^R/Carb^R$. | This study. |
| pBBR1MCS4-*tssA2-HA* | pBBR1MCS4 encoding C-terminal HA tagged *tssA2* (PA1656), $Amp^R/Carb^R$. | This study. |
| pBBR1MCS4-*tssA3-HA* | pBBR1MCS4 encoding C-terminal HA tagged *tssA3* (PA2360), $Amp^R/Carb^R$. | This study. |

| | | |
|---|---|---|
| pBBR1MCS4-*tssA1*$_{Nt1}$-*tssA2*$_{CTD}$-*HA* | pBBR1MCS4 encoding C-terminal HA tagged *tssA1* Nt1 (1-275) *tssA2* Nt2+CTD (388-518) chimera, $Amp^R$/$Carb^R$. | This study. |
| pBBR1MCS4-*tssA1*$_{Nt1}$-*tssA2*$_{Nt2+CTD}$-*HA* | pBBR1MCS4 encoding C-terminal HA tagged *tssA1* Nt1 (1-275) *tssA2* Nt2+CTD (222-518) chimera, $Amp^R$/$Carb^R$. | This study. |
| pBBR1MCS4-*tssA1*$_{Nt1}$-*tssA2*$_{Nt2+CTD}$-*HA* | pBBR1MCS4 encoding C-terminal HA tagged *tssA1* Nt1 (1-275) *tssA2* Nt2+CTD (222-518) chimera, $Amp^R$/$Carb^R$. | This study. |
| pBBR1MCS4-*tssA1*$_{Nt1}$-*tssA3*$_{CTD}$-*HA* | pBBR1MCS4 encoding C-terminal HA tagged *tssA1* Nt1 (1-275) *tssA3* CTD (296-366) chimera, $Amp^R$/$Carb^R$. | This study. |
| pBBR1MCS4-*tssA2*$_{Nt1}$-*tssA1*$_{CTD}$-*HA* | pBBR1MCS4 encoding C-terminal HA tagged *tssA2* Nt1 (1-192) *tssA1* CTD (246-344) chimera, $Amp^R$/$Carb^R$. | This study. |
| pBBR1MCS4-*tssA2*$_{Nt1}$-*tssA3*$_{CTD}$-*HA* | pBBR1MCS4 encoding C-terminal HA tagged *tssA2* Nt1 (1-222) *tssA3* CTD (296-366) chimera, $Amp^R$/$Carb^R$. | This study. |
| pBBR1MCS4-*tssA3*$_{Nt1}$-*tssA2*$_{CTD}$-*HA* | pBBR1MCS4 encoding C-terminal HA tagged *tssA3* Nt1 (1-262) *tssA2* CTD (375-518) chimera, $Amp^R$/$Carb^R$. | This study. |
| pBBR1MCS4-*tssA3*$_{Nt1}$-*tssA2*$_{Nt2+CTD}$-*HA* | pBBR1MCS4 encoding C-terminal HA tagged *tssA3* Nt1 (1-262) *tssA2* Nt2+CTD (192-518) chimera, $Amp^R$/$Carb^R$. | This study. |
| pBBR1MCS4-*tssA1*-*tssA3*$_{hairpin}$-*HA* | pBBR1MCS4 encoding C-terminal HA tagged *tssA1* with hairpin region (F169-L172) replaced with *tssA3* hairpin region (A174-Q191), $Amp^R$/$Carb^R$. | This study. |
| pBBR1MCS4-*tssA1*-*tssA3*$_{loop}$-*HA* | pBBR1MCS4 encoding C-terminal HA tagged *tssA1* with loop region (Q37-E53) replaced with *tssA3* loop region (D46- D62), $Amp^R$/$Carb^R$. | This study. |
| pBBR1MCS4-*tssA3*-*tssA1*$_{hairpin}$-*HA* | pBBR1MCS4 encoding C-terminal HA tagged *tssA3* with hairpin region (A174-Q191) replaced with *tssA1* hairpin region (F169-L172), $Amp^R$/$Carb^R$. | This study. |
| pBBR1MCS4-*tssA3*-*tssA1*$_{loop}$-*HA* | pBBR1MCS4 encoding C-terminal HA tagged *tssA3* with loop region (D46-D62) replaced with *tssA1* loop region (Q37- E53), $Amp^R$/$Carb^R$. | This study. |
| pBBR1MCS4-*tssA3*-*tssA1*$_{hairpin\ and\ loop}$-*HA* | pBBR1MCS4 encoding C-terminal HA tagged *tssA3* with hairpin (A174- Q191) and loop (D46-D62) regions replaced with *tssA1* hairpin (F169-L172) and loop (Q37-E53) regions, $Amp^R$/$Carb^R$. | This study. |

| | | |
|---|---|---|
| pBBR1MCS4-*tssA2*$_{Nt1+Nt2}$-*tssA1*$_{CTD}$ -*HA* | pBBR1MCS4 encoding C-terminal HA tagged *tssA2*$_{Nt1+Nt2}$ (1-388) and *tssA1*$_{CTD}$ (275-344) chimera, $Amp^R$/$Carb^R$ | This study. |
| pBBR1MCS4-*tssA2*$_{Nt1+Nt2}$-*tssA3*$_{CTD}$ -*HA* | pBBR1MCS4 encoding C-terminal HA tagged *tssA2*$_{Nt1+Nt2}$ (1-388) and *tssA3*$_{CTD}$ (296-366) chimera, $Amp^R$/$Carb^R$ | This study. |
| pBBR1MCS4-*tssA1*$^{PP}$ -*HA* | pBBR1MCS4 encoding C-terminal HA tagged full length *P. putida tssA1* (PP3088), $Amp^R$/$Carb^R$ | This study. |
| pBBR1MCS4-*tssA1*$^{PA}_{Nt1}$-*tssA1*$^{PP}_{CTD}$ -*HA* | pBBR1MCS4 encoding C-terminal HA tagged *tssA1*$^{PA}_{Nt1}$ (1-275) and *tssA1*$^{PP}_{CTD}$ (22-361) chimera, $Amp^R$/$Carb^R$ | This study. |
| pBBR1MCS4-*tssA1*$^{PP}_{Nt1}$-*tssA1*$^{PA}_{CTD}$ -*HA* | pBBR1MCS4 encoding C-terminal HA tagged *tssA1*$^{PP}_{Nt1}$ (1-259) and *tssA1*$^{PA}_{CTD}$ (246-344) chimera, $Amp^R$/$Carb^R$ | This study. |
| pRL662-GFP | Broad host range vector producing GFP under a constitutive $P_{LAC}$ promoter, pBBR1 ori, $Gm^R$. | (Howard *et al.,* 2021)[11] |
| pETDuet-1 | High expression vector with two multiple cloning sites, both with IPTG inducible T7 promoter, pBR322 ori, $Amp^R$. | EMD Biosciences |
| pETDuet-1-*Strep-tssA1 CTD* | pETDuet-1 encoding N-terminal Strep tagged *tssA1* (PA0082) CTD residues 275-344, $Amp^R$. | This study. |
| pETDuet-1-*Strep-tssA3 CTD* | pETDuet-1 encoding N-terminal Strep tagged *tssA3* (PA2360) CTD residues 296-366, $Amp^R$. | This study. |
| | **Chromosomal mutagenesis vectors** | |
| pKNG101 | Non-replicative suicide vector to alter *P. aeruginosa* chromosome, *ori6K*, *mobRK2*, *sacB*, $Sm^R$. | (Kaniga, Delor and Cornelis, 1991)[12] |
| pKNG101-Δ*rsmN* | pKNG101 suicide plasmid to delete *rsmN/F* (PA5183.1), $Sm^R$. | This study. |
| pKNG101-Δ*retS* | pKNG101 suicide plasmid to delete *retS* (PA4856), $Sm^R$. | This study. |
| pKNG101-Δ*tssA1* | pKNG101 suicide plasmid to delete *tssA1* (PA0082), $Sm^R$. | This study. |
| pKNG101-Δ*tssA2* | pKNG101 suicide plasmid to delete *tssA2* (PA1656), $Sm^R$. | This study. |
| pKNG101-Δ*tssA3* | pKNG101 suicide plasmid to delete *tssA3* (PA2360), $Sm^R$. | This study. |
| pKNG101-*tssB1-mScarlet-I* | pKNG101 suicide plasmid to introduce *mScarlet-I* at the C-terminus of the *tssB1* gene (PA0083), $Sm^R$. | This study. |
| pKNG101-*tssB2-mScarlet-I* | pKNG101 suicide plasmid to introduce *mScarlet-I* at the C-terminus of the *tssB2* gene (PA1657), $Sm^R$. | This study. |
| pKNG101-*tssB3-mScarlet-I* | pKNG101 suicide plasmid to introduce *mScarlet-I* at the C-terminus of the *tssB3* gene (PA2365), $Sm^R$. | This study. |

**Supplementary Table 4.** Antibodies used in this study.

| **Antibody** | **Host** | **Sera** | **Dilution** | **Source** |
|---|---|---|---|---|
| anti-Hcp1 | Rabbit | Polyclonal | 1:500 | (Hachani *et al*., 2011)[13] |
| anti-Hcp2 | Rabbit | Polyclonal | 1:500 | (Jones *et al.,* 2014)[14] |
| anti-RpoB | Mouse | Monoclonal | 1:1000 | Neoclone |
| anti-HA | Rabbit | Monoclonal | 1:1000 | Sigma |
| HRP-conjugated anti-Mouse | Rabbit | Polyclonal | 1:5000 | Sigma |
| HRP-conjugated anti-Rabbit | Goat | Polyclonal | 1:5000 | Sigma |

**Supplementary References**


1 Karimova, G., Pidoux, J., Ullmann, A. & Ladant, D. A bacterial two-hybrid system based on a reconstituted signal transduction pathway. *Proc Natl Acad Sci U S A* **95**, 5752-5756, doi:10.1073/pnas.95.10.5752 (1998).

2 Herrero, M., de Lorenzo, V. & Timmis, K. N. Transposon vectors containing non-antibiotic resistance selection markers for cloning and stable chromosomal insertion of foreign genes in gram-negative bacteria. *J Bacteriol* **172**, 6557-6567, doi:10.1128/jb.172.11.6557-6567.1990 (1990).

3 Figurski, D. H. & Helinski, D. R. Replication of an origin-containing derivative of plasmid RK2 dependent on a plasmid function provided in trans. *Proc Natl Acad Sci U S A* **76**, 1648-1652, doi:10.1073/pnas.76.4.1648 (1979).

4 Allsopp, L. P. *et al.* RsmA and AmrZ orchestrate the assembly of all three type VI secretion systems in *Pseudomonas aeruginosa*. *Proc Natl Acad Sci U S A* **114**, 7707-7712, doi:10.1073/pnas.1700286114 (2017).

5 Rudzite, M., Subramoni, S., Endres, R. G. & Filloux, A. Effectiveness of *Pseudomonas aeruginosa* type VI secretion system relies on toxin potency and type IV pili-dependent interaction. *PLoS Pathog* **19**, e1011428, doi:10.1371/journal.ppat.1011428 (2023).

6 Karimova, G., Ullmann, A. & Ladant, D. Protein-protein interaction between *Bacillus stearothermophilus* tyrosyl-tRNA synthetase subdomains revealed by a bacterial two-hybrid system. *J Mol Microbiol Biotechnol* **3**, 73-82 (2001).

7 Lossi, N. S. *et al.* The archetype *Pseudomonas aeruginosa* proteins TssB and TagJ form a novel subcomplex in the bacterial type VI secretion system. *Mol Microbiol* **86**, 437-456, doi:10.1111/j.1365-2958.2012.08204.x (2012).

8 Planamente, S. *et al.* TssA forms a gp6-like ring attached to the type VI secretion sheath. *EMBO J* **35**, 1613-1627, doi:10.15252/embj.201694024 (2016).

9 Lossi, N. S. *et al.* The HsiB1C1 (TssB-TssC) complex of the *Pseudomonas aeruginosa* type VI secretion system forms a bacteriophage tail sheathlike structure. *J Biol Chem* **288**, 7536-7548, doi:10.1074/jbc.M112.439273 (2013).

10 Kovach, M. E. *et al.* Four new derivatives of the broad-host-range cloning vector pBBR1MCS, carrying different antibiotic-resistance cassettes. *Gene* **166**, 175-176, doi:10.1016/0378-1119(95)00584-1 (1995).

11 Howard, S. A. *et al.* The Breadth and Molecular Basis of Hcp-Driven Type VI Secretion System Effector Delivery. *mBio* **12**, e0026221, doi:10.1128/mBio.00262-21 (2021).

12 Kaniga, K., Delor, I. & Cornelis, G. R. A wide-host-range suicide vector for improving reverse genetics in gram-negative bacteria: inactivation of the blaA gene of *Yersinia enterocolitica*. *Gene* **109**, 137-141, doi:10.1016/0378-1119(91)90599-7 (1991).

13 Hachani, A. *et al.* Type VI secretion system in *Pseudomonas aeruginosa*: secretion and multimerization of VgrG proteins. *J Biol Chem* **286**, 12317-12327, doi:10.1074/jbc.M110.193045 (2011).

14 Jones, C., Hachani, A., Manoli, E. & Filloux, A. An rhs gene linked to the second type VI secretion cluster is a feature of the *Pseudomonas aeruginosa* strain PA14. *J Bacteriol* **196**, 800-810, doi:10.1128/JB.00863-13 (2014).
