# Supplementary material for: Functionality of chimeric TssA proteins in the type VI secretion system reveals sheath docking specificity within their N-terminal domains
Source: Nat Commun. 2024 May 20;15:4283. doi: 10.1038/s41467-024-48487-8 (PMC11106082; doi:10.1038/s41467-024-48487-8)
Supplement: Supplementary file 3 — Reporting Summary [file 41467_2024_48487_MOESM3_ESM.pdf]

nature portfolio

Corresponding author(s): Alain Filloux

Last updated by author(s): Apr 19, 2024

Reporting Summary

Nature Portfolio wishes to improve the reproducibility of the work that we publish. This form provides structure for consistency and transparency in reporting. For further information on Nature Portfolio policies, see our Editorial Policies and the Editorial Policy Checklist.

Statistics

For all statistical analyses, confirm that the following items are present in the figure legend, table legend, main text, or Methods section.

n/a	Confirmed	
☐	☒	The exact sample size (*n*) for each experimental group/condition, given as a discrete number and unit of measurement
☐	☒	A statement on whether measurements were taken from distinct samples or whether the same sample was measured repeatedly
☐	☒	The statistical test(s) used AND whether they are one- or two-sided<br>*Only common tests should be described solely by name; describe more complex techniques in the Methods section.*
☒	☐	A description of all covariates tested
☐	☒	A description of any assumptions or corrections, such as tests of normality and adjustment for multiple comparisons
☐	☒	A full description of the statistical parameters including central tendency (e.g. means) or other basic estimates (e.g. regression coefficient) AND variation (e.g. standard deviation) or associated estimates of uncertainty (e.g. confidence intervals)
☐	☒	For null hypothesis testing, the test statistic (e.g. *F*, *t*, *r*) with confidence intervals, effect sizes, degrees of freedom and *P* value noted<br>*Give P values as exact values whenever suitable.*
☒	☐	For Bayesian analysis, information on the choice of priors and Markov chain Monte Carlo settings
☒	☐	For hierarchical and complex designs, identification of the appropriate level for tests and full reporting of outcomes
☒	☐	Estimates of effect sizes (e.g. Cohen's *d*, Pearson's *r*), indicating how they were calculated

Our web collection on statistics for biologists contains articles on many of the points above.

Software and code

Policy information about availability of computer code

Data collection	serialEM 4.0.10
Data analysis	ChimeraX v1.5, CryoSparc v4.1, RosettaCM 4.20, Clustal Omega 1.2.4, SWISS-MODEL, GetArea, ZDock server 3.0.2, WinCoot 0.9.4.1, DaliLite.v5, GraphPad Prism v9.4.0, ZEISS ZEN 3.4, FIJI v2.0.0/1.53c, Image Lab v6.1.0

For manuscripts utilizing custom algorithms or software that are central to the research but not yet described in published literature, software must be made available to editors and reviewers. We strongly encourage code deposition in a community repository (e.g. GitHub). See the Nature Portfolio guidelines for submitting code & software for further information.

Data

Policy information about availability of data

All manuscripts must include a data availability statement. This statement should provide the following information, where applicable:

- Accession codes, unique identifiers, or web links for publicly available datasets
- A description of any restrictions on data availability
- For clinical datasets or third party data, please ensure that the statement adheres to our policy

All data are available in the main text and supplementary materials, with uncropped scans of all gels and blots, as well as raw data for all graphs, provided in source data files. The structural data of the contracted P. aeruginosa contracted sheath used in this study are available in the Protein Data Bank (PDB) under accession code 5N8N [https://doi.org/10.2210/pdb5N8N/pdb]. Correspondence and requests for material should be addressed to A.F.

Research involving human participants, their data, or biological material

Policy information about studies with human participants or human data. See also policy information about sex, gender (identity/presentation), and sexual orientation and race, ethnicity and racism.

Reporting on sex and gender	N/A
Reporting on race, ethnicity, or other socially relevant groupings	N/A
Population characteristics	N/A
Recruitment	N/A
Ethics oversight	N/A

Note that full information on the approval of the study protocol must also be provided in the manuscript.

Field-specific reporting

Please select the one below that is the best fit for your research. If you are not sure, read the appropriate sections before making your selection.

[x] Life sciences [ ] Behavioural & social sciences [ ] Ecological, evolutionary & environmental sciences

For a reference copy of the document with all sections, see nature.com/documents/nr-reporting-summary-flat.pdf

Life sciences study design

All studies must disclose on these points even when the disclosure is negative.

Sample size	No sample size calculation was performed. Sample sizes were selected based on previous experience and published studies. For microscopy datasets, the sample comprised cells counted from three fields of view for at least two experiments.
Data exclusions	No data were excluded.
Replication	All experiments, including secretion and competition assays, bacterial two-hybrid and microscopy, were repeated a minimum of two or three times. For quantitative data all repeats are presented, for qualitative data a representative repeat is presented. Appropriate tests were carried out to determine the statistical significance of the data. All attempts at replication were successful.
Randomization	Randomization was not relevant to this study. Reproducibility was demonstrated through replication carried out on different days.
Blinding	Blinding was not relevant to this study. All samples for a given experiment were processed in parallel. Much of the data was quantitative, and as such, not open to bias in interpretation of the results.

Reporting for specific materials, systems and methods

We require information from authors about some types of materials, experimental systems and methods used in many studies. Here, indicate whether each material, system or method listed is relevant to your study. If you are not sure if a list item applies to your research, read the appropriate section before selecting a response.

Materials & experimental systems

n/a	Involved in the study	
[ ]	[x]	Antibodies
[x]	[ ]	Eukaryotic cell lines
[x]	[ ]	Palaeontology and archaeology
[x]	[ ]	Animals and other organisms
[x]	[ ]	Clinical data
[x]	[ ]	Dual use research of concern
[x]	[ ]	Plants

Methods

n/a	Involved in the study	
[x]	[ ]	ChIP-seq
[x]	[ ]	Flow cytometry
[x]	[ ]	MRI-based neuroimaging

Antibodies

Antibodies used	anti-Hcp1, Eurogentec<br>anti-Hcp2, Eurogentec

	anti-RpoB, Neoclone, catalogue number 663903<br>anti-HA, Sigma Aldrich, catalogue number H6908<br>HRP-conjugated anti-Mouse, Sigma Aldrich, catalogue number AB6789<br>HRP-conjugated anti-Rabbit, Sigma Aldrich, catalogue number A0545
Validation	The use of anti-Hcp1, anti-Hcp2, and anti-RpoB has been validated in P. aeruginosa in previous reports. For anti-Hcp1 in Type VI secretion system in Pseudomonas aeruginosa: secretion and multimerization of VgrG proteins (Hachani et al., 2011), for anti-Hcp2 in An rhs gene linked to the second type VI secretion cluster is a feature of the Pseudomonas aeruginosa strain PA14 (Jones et al., 2014) and for anti-RpoB in LadS is a calcium-responsive kinase that induces acute-to-chronic virulence switch in Pseudomonas aeruginosa (Broder at al., 2016).

Plants

Seed stocks	N/A
Novel plant genotypes	N/A
Authentication	N/A
