# Supplementary material for: Functionality of chimeric TssA proteins in the type VI secretion system reveals sheath docking specificity within their N-terminal domains
Source: Nat Commun. 2024 May 20;15:4283. doi: 10.1038/s41467-024-48487-8 (PMC11106082; doi:10.1038/s41467-024-48487-8)

**Figure 2a**

Hcp1 cell and supernatant


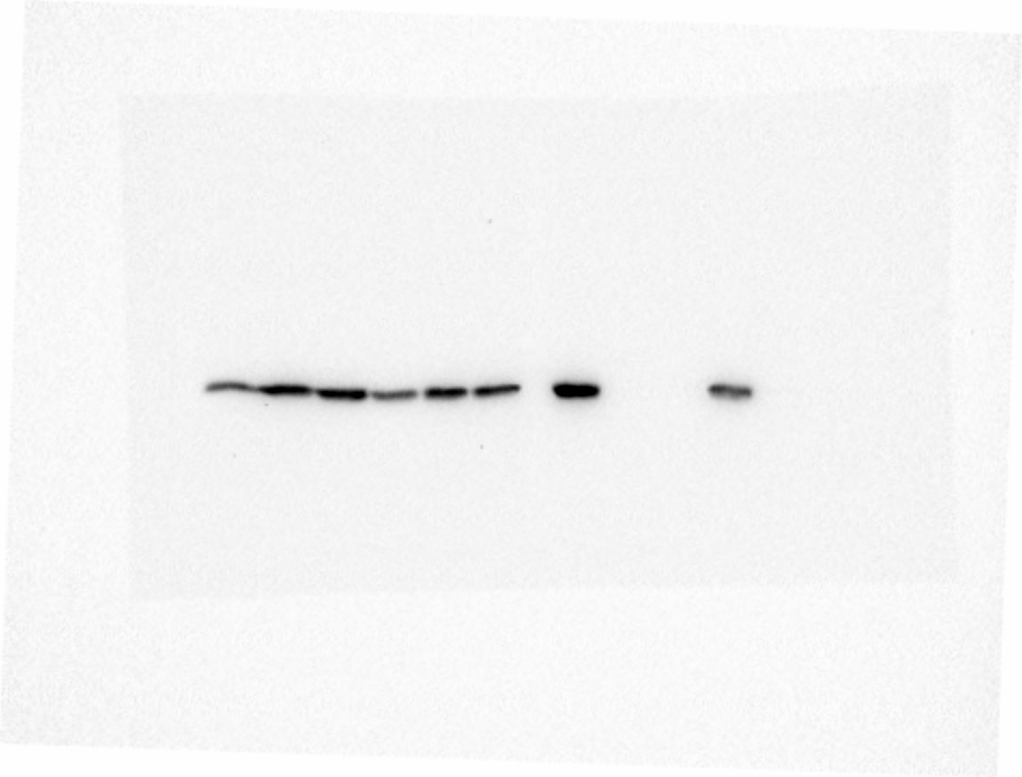


RpoB cell and supernatant


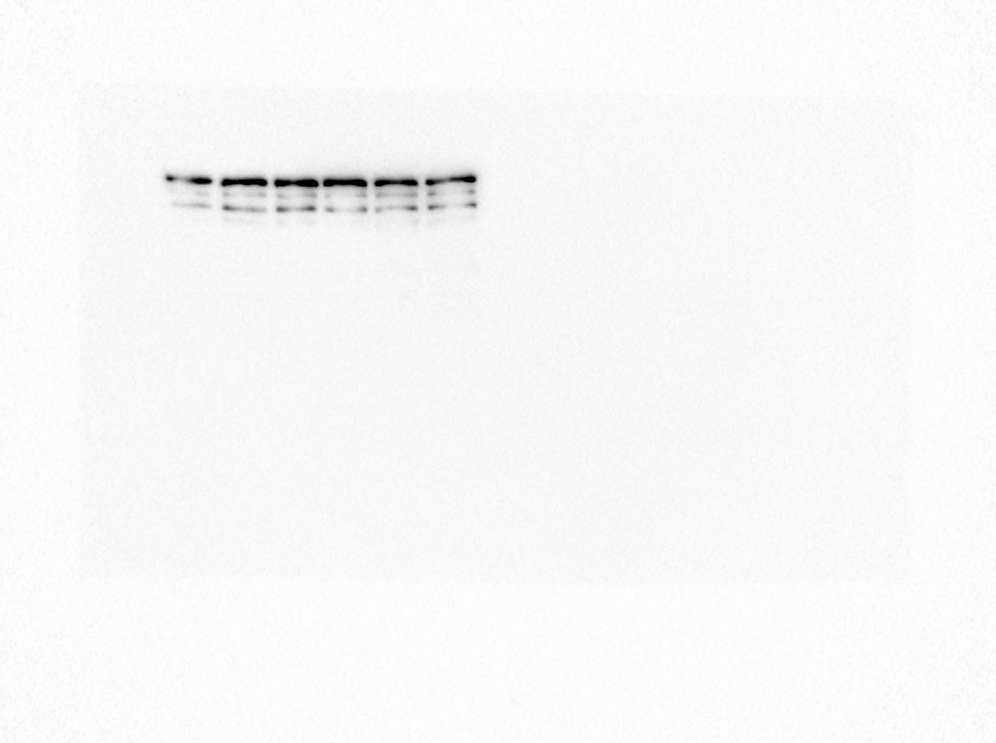


**Figure 2c**

Hcp2 cell


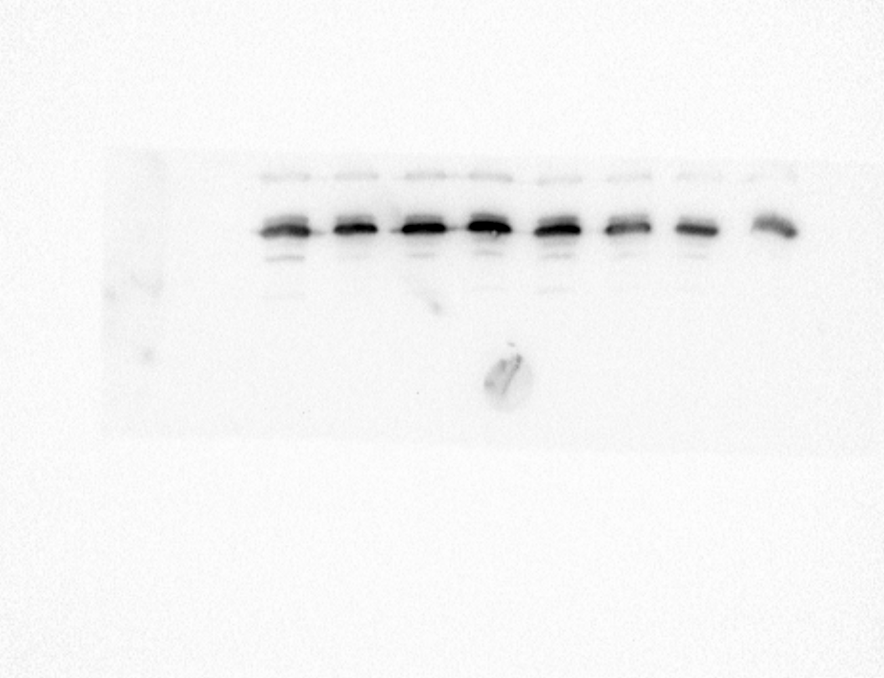


Hcp2 supernatant


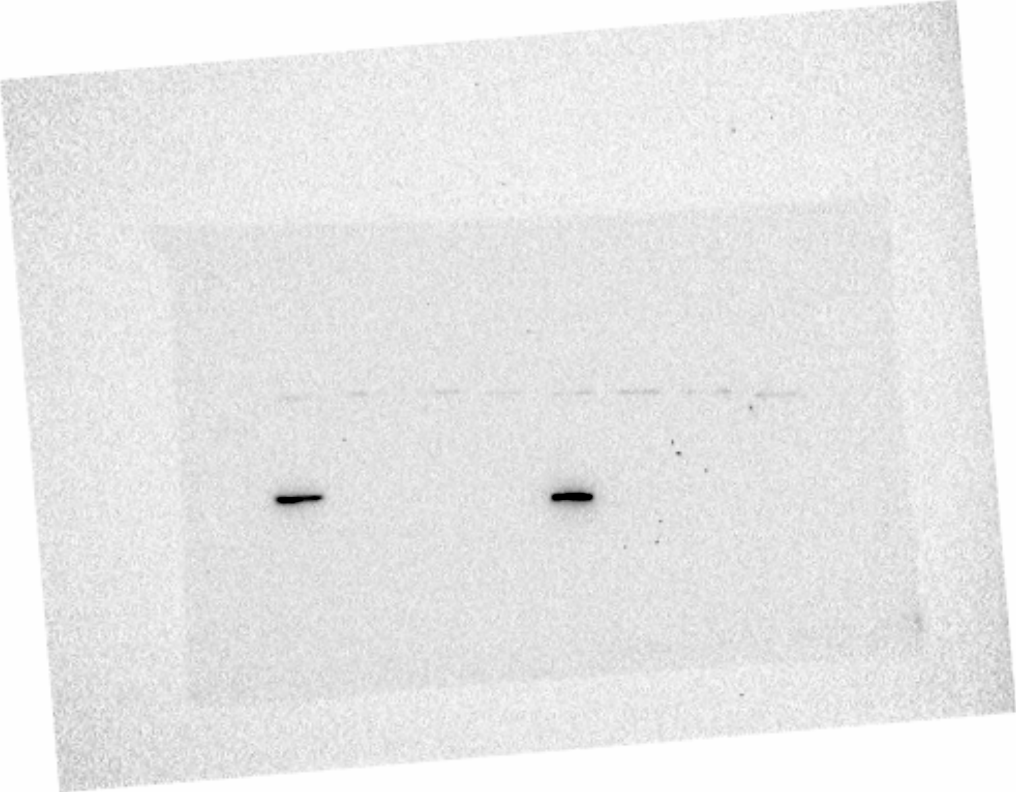


RpoB cell


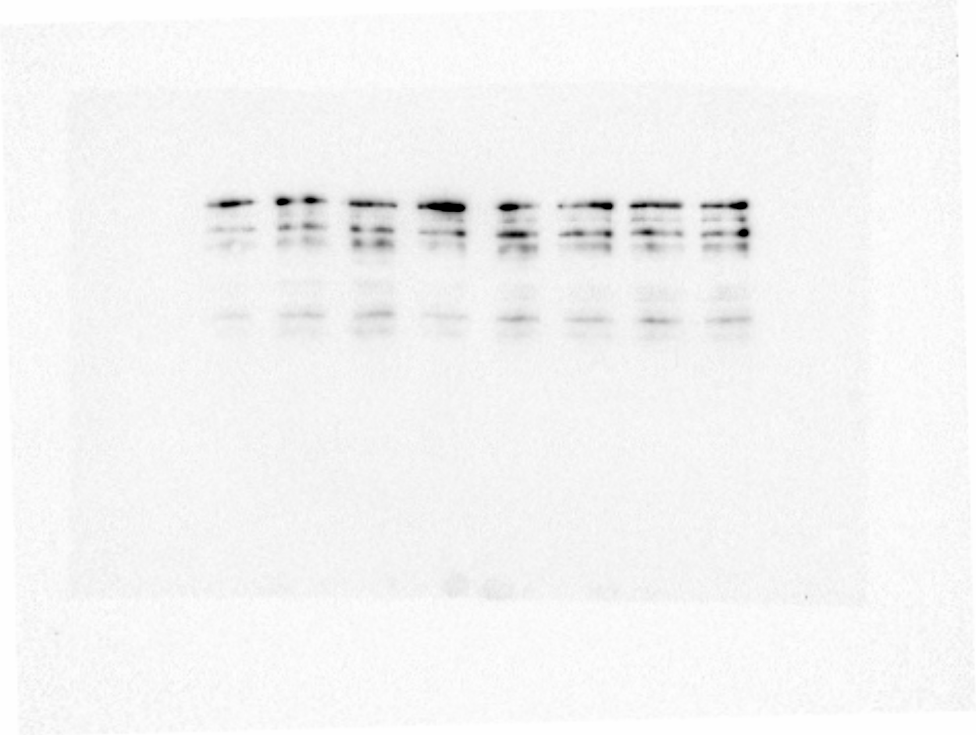


RpoB supernatant


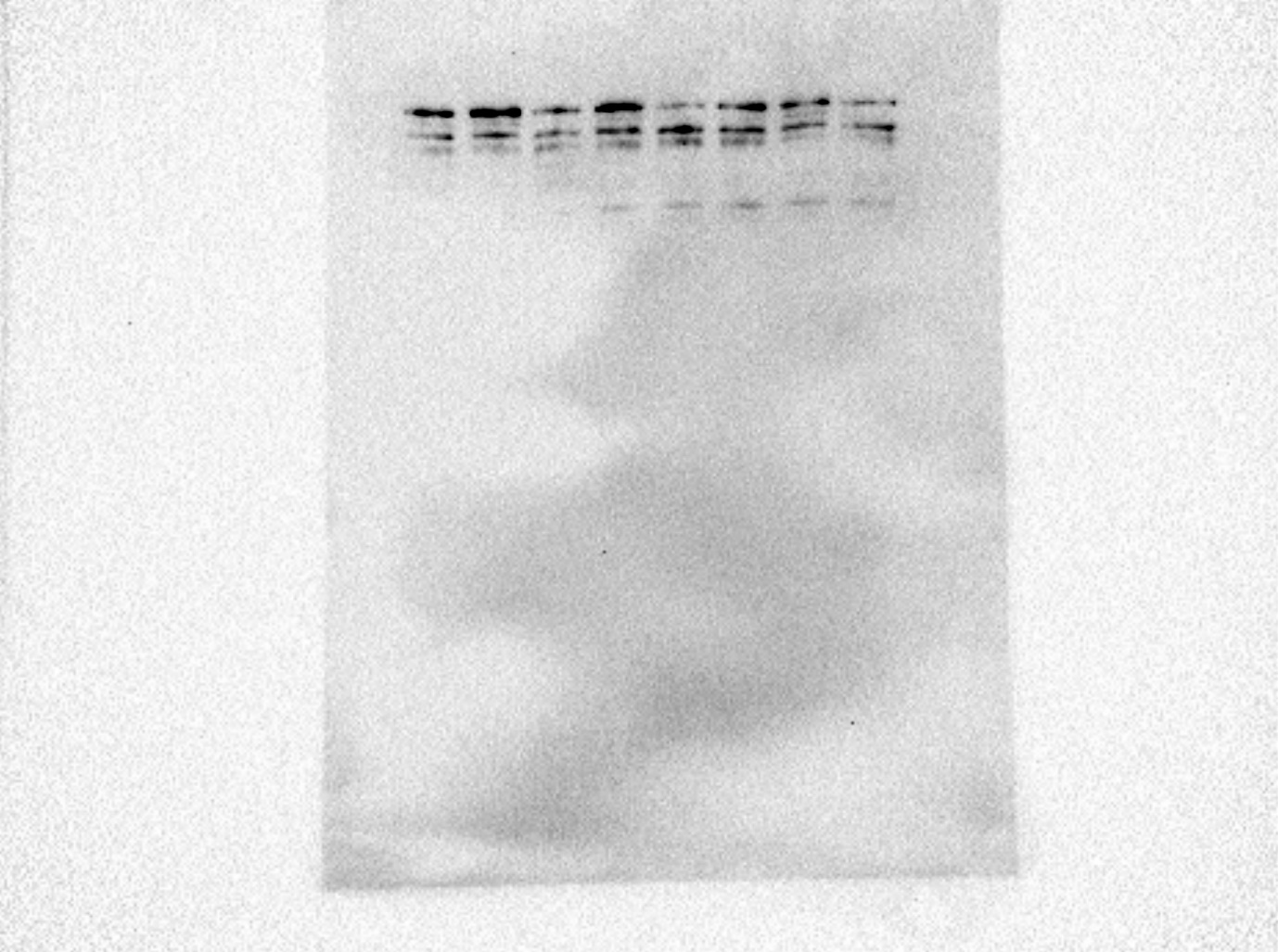

Supplement: Supplementary file 4 — Source data [file 41467_2024_48487_MOESM4_ESM.zip › Source Data/Source Data Figure 2.docx]
