# Supplementary material for: Functionality of chimeric TssA proteins in the type VI secretion system reveals sheath docking specificity within their N-terminal domains
Source: Nat Commun. 2024 May 20;15:4283. doi: 10.1038/s41467-024-48487-8 (PMC11106082; doi:10.1038/s41467-024-48487-8)
Supplement: Supplementary file 4 — Source data [file 41467_2024_48487_MOESM4_ESM.zip › Source Data/Source Data Supplementary Figure 13.docx]

**Supplementary Figure 13a**

*tssA1-tssA3_hairpin_-HA, tssA1-tssA3_loop_-HA,* anti-HA

*tssA1-tssA3_hairpin_-HA, tssA1-tssA3_loop_-HA,* anti-RpoB

*tssA3-tssA1_hairpin_-HA, tssA3-tssA1_loop_-HA, tssA3-tssA1_hairpin+loop_-HA* anti-HA

*tssA3-tssA1_hairpin_-HA, tssA3-tssA1_loop_-HA, tssA3-tssA1_hairpin+loop_-HA* anti-RpoB
