# Supplementary material for: Functionality of chimeric TssA proteins in the type VI secretion system reveals sheath docking specificity within their N-terminal domains
Source: Nat Commun. 2024 May 20;15:4283. doi: 10.1038/s41467-024-48487-8 (PMC11106082; doi:10.1038/s41467-024-48487-8)
Supplement: Supplementary file 4 — Source data [file 41467_2024_48487_MOESM4_ESM.zip › Source Data/Source Data Supplementary Figure 6.docx]

**Supplementary Figure 6**

EV and *tssA1_Nt1_tssA2_CTD_*-HA, anti-HA


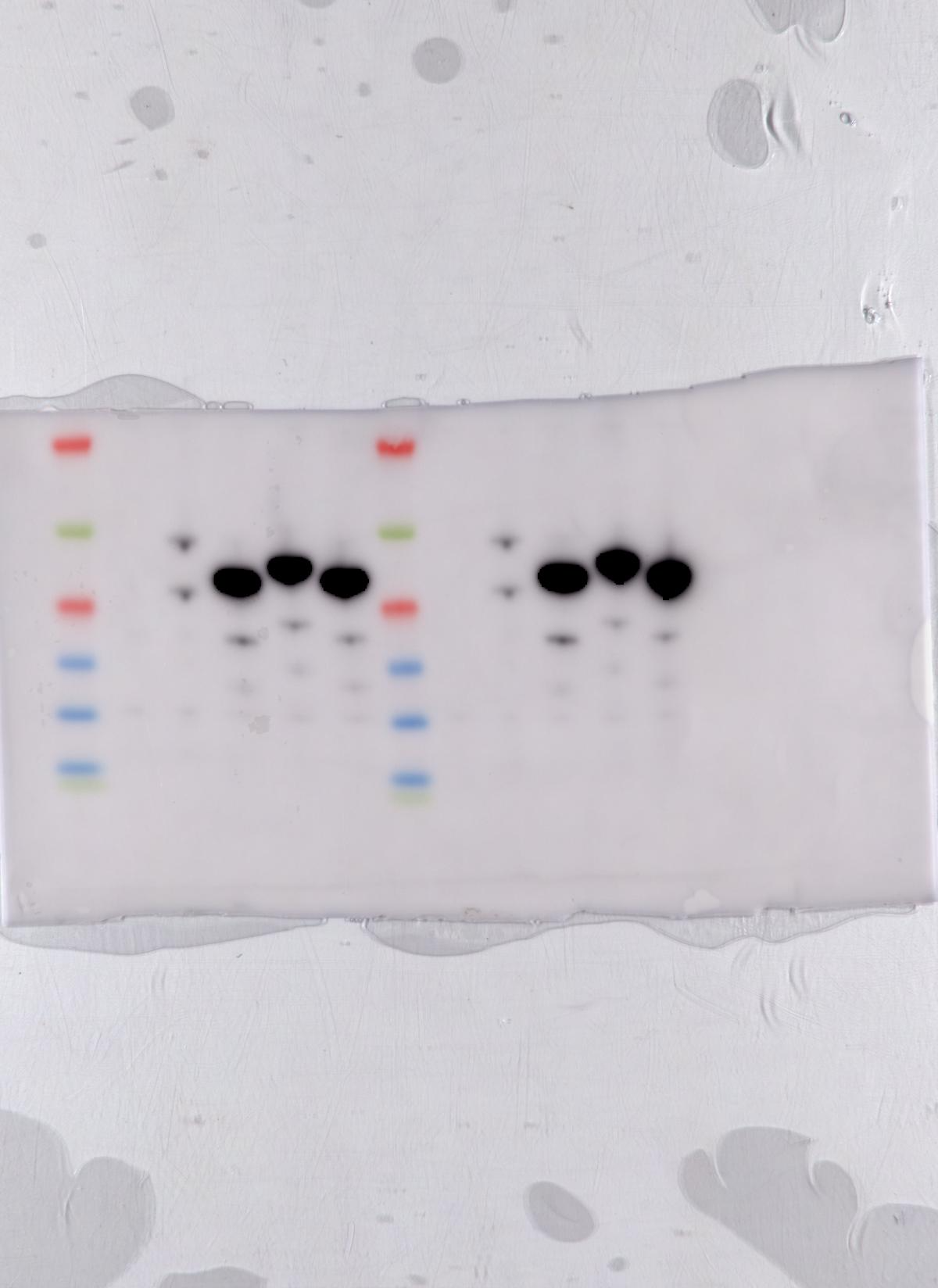


EV and *tssA1_Nt1_tssA2_CTD_*-HA, anti-RpoB


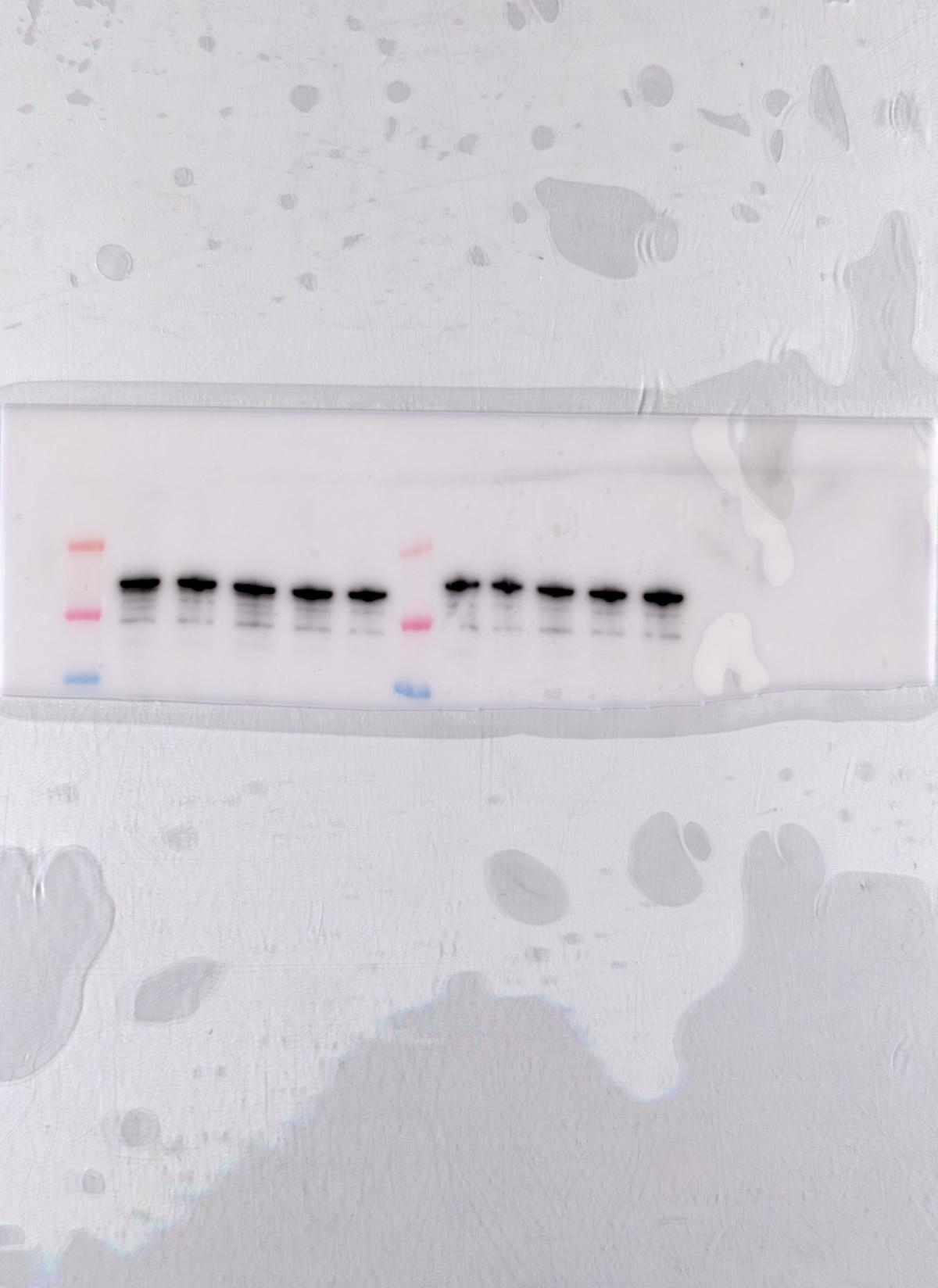


*tssA1_Nt1_tssA2_Nt1+CTD_*-HA, anti-HA


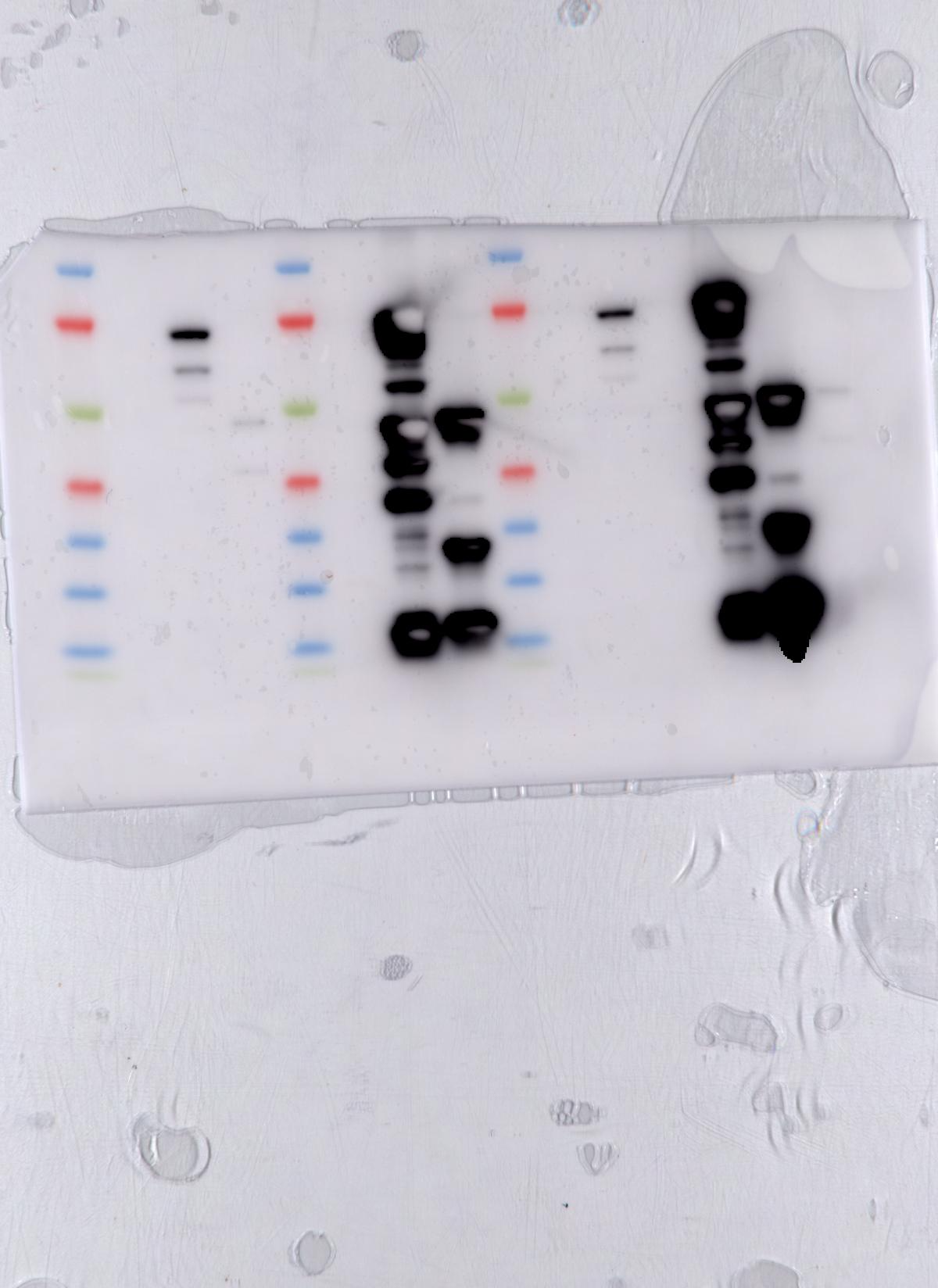


*tssA1_Nt1_tssA2_Nt1+CTD_*-HA, anti-RpoB


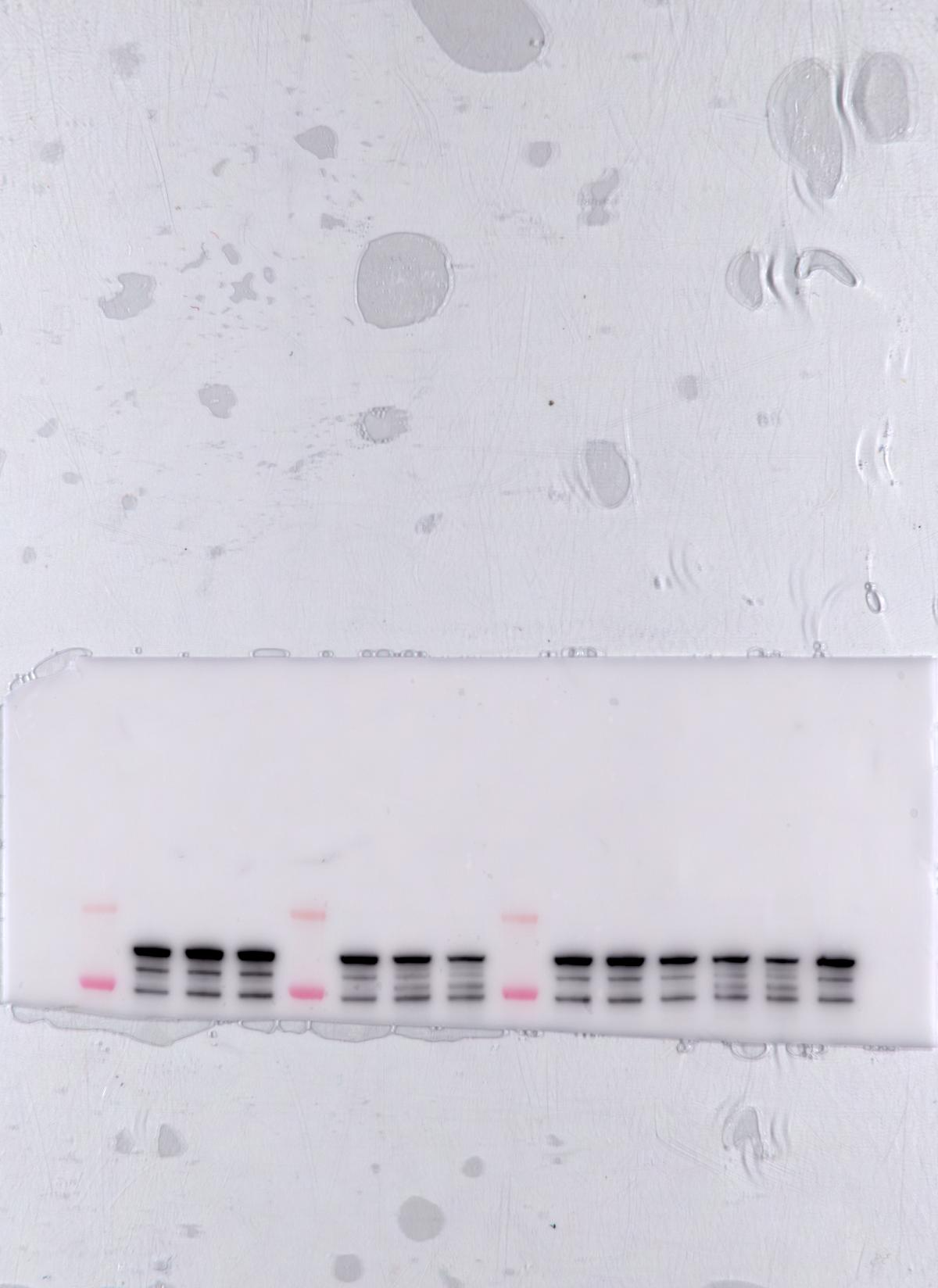


*tssA1_Nt1_tssA3_CTD_-HA,* anti-HA

*tssA1_Nt1_tssA3_CTD_-HA,* anti-RpoB

*tssA2_Nt1_tssA1_CTD_*-HA, anti-HA


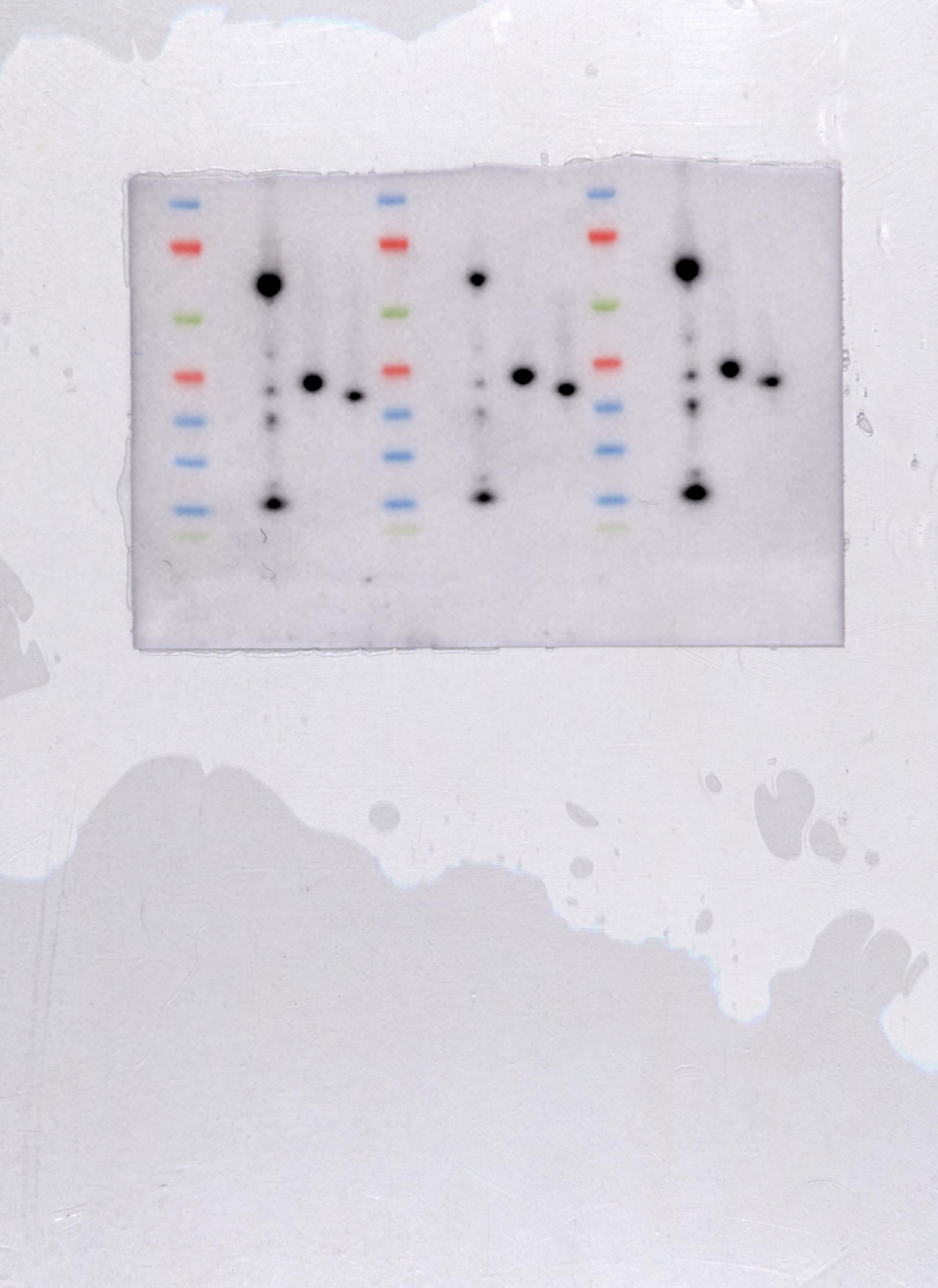


*tssA2_Nt1_tssA1_CTD_*-HA, anti-RpoB


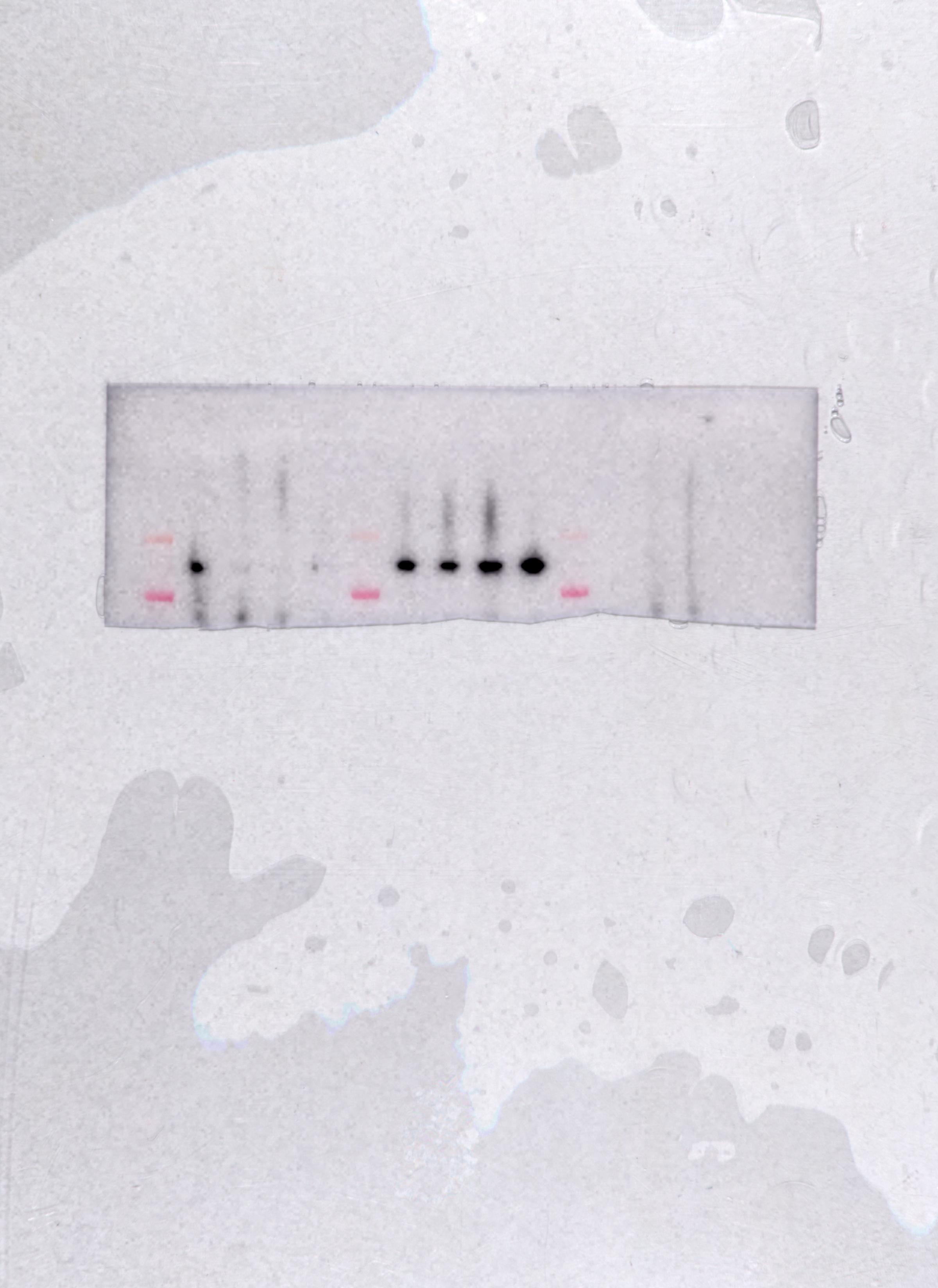


*tssA2_Nt1_tssA3_CTD_-HA*, anti-HA


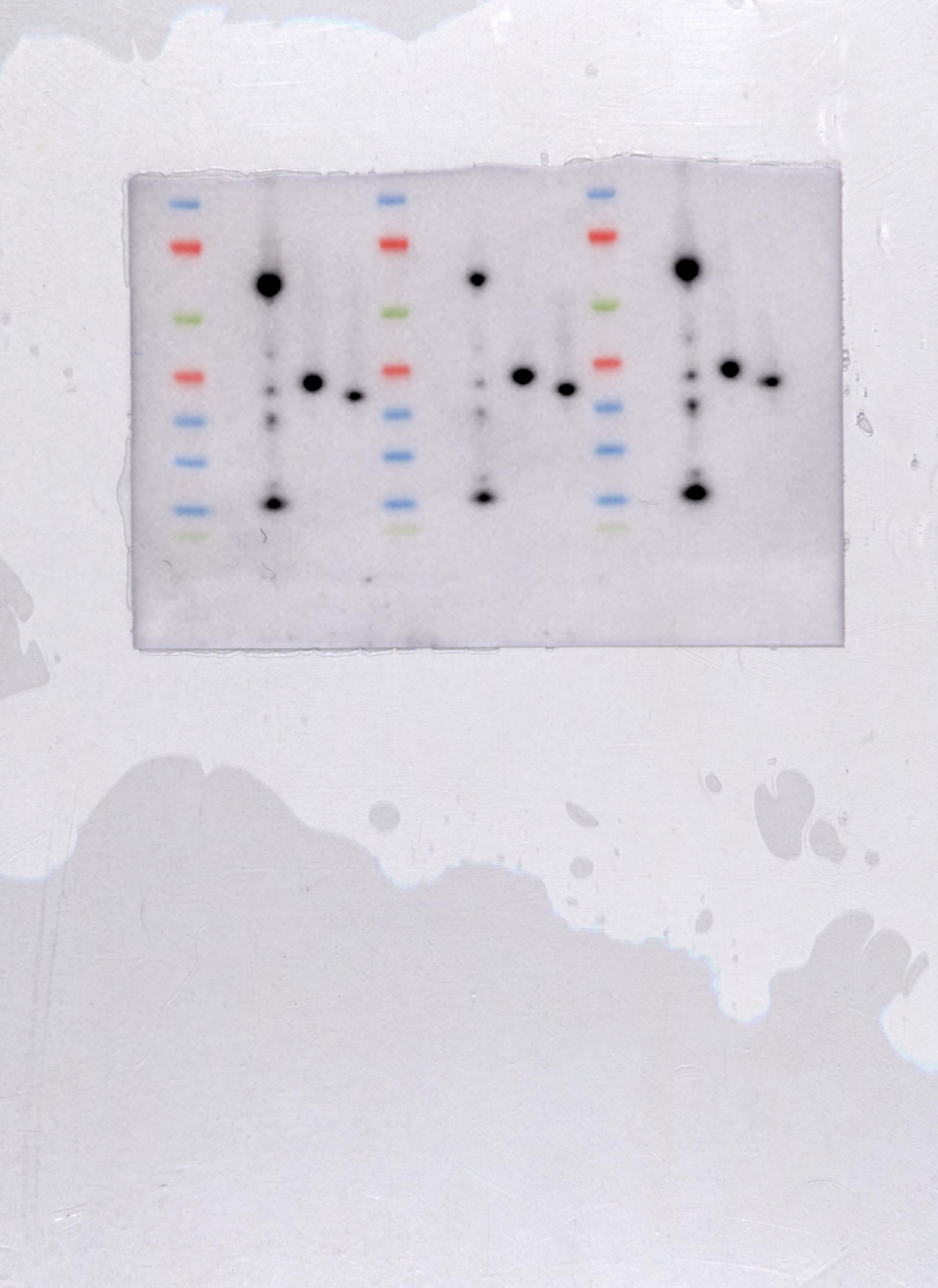


*tssA2_Nt1_tssA3_CTD_-HA*, anti-RpoB


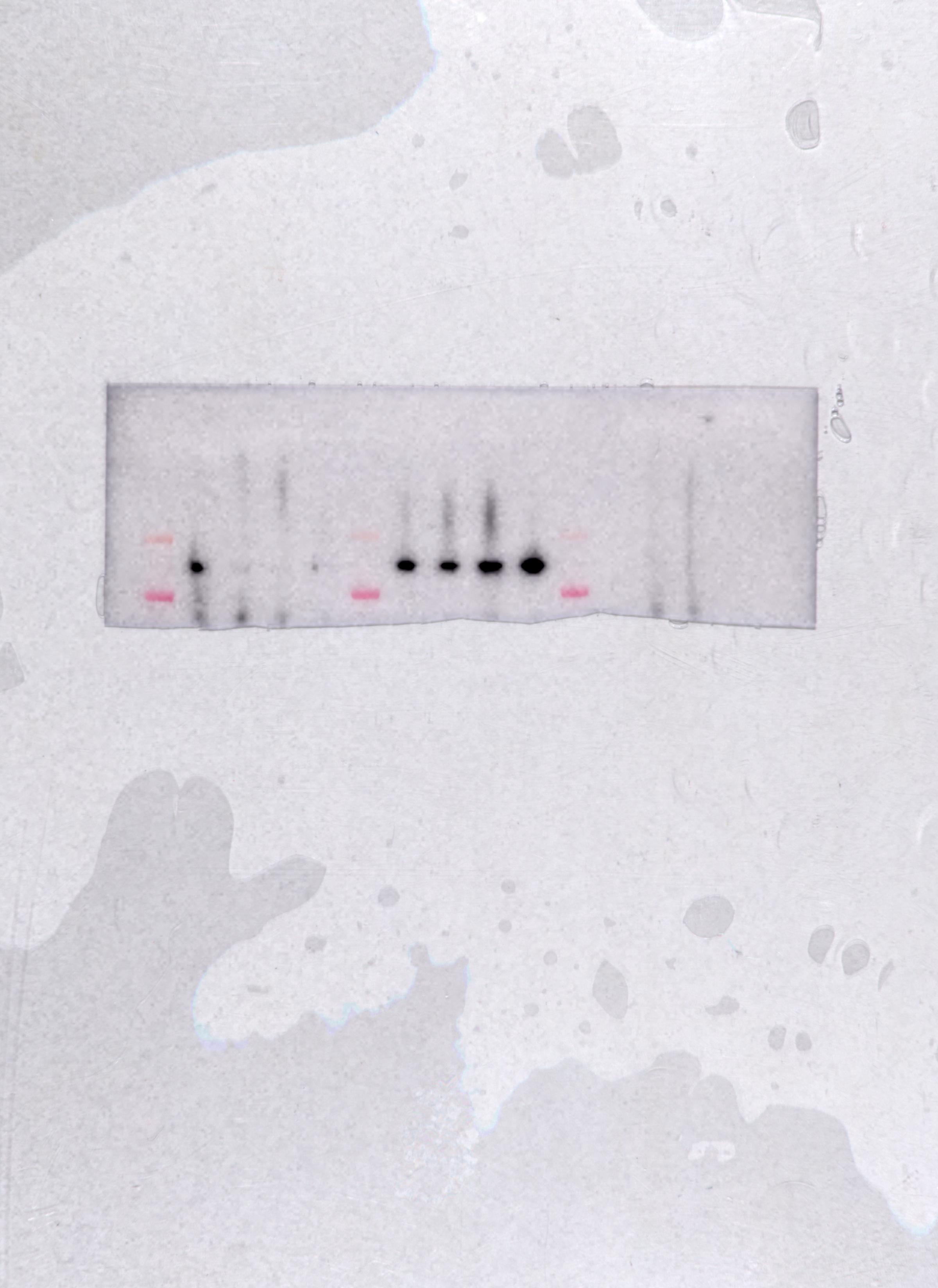


*tssA3_Nt1_tssA2_Nt2+CTD_,* anti-HA

*tssA3_Nt1_tssA2_Nt2+CTD_,* anti-RpoB

*tssA3_Nt1_tssA2_CTD_,* anti-HA

*tssA3_Nt1_tssA2_CTD_,* anti-RpoB
