# Supplementary material for: Functionality of chimeric TssA proteins in the type VI secretion system reveals sheath docking specificity within their N-terminal domains
Source: Nat Commun. 2024 May 20;15:4283. doi: 10.1038/s41467-024-48487-8 (PMC11106082; doi:10.1038/s41467-024-48487-8)

Supplementary Figure 7b

*tssA1^PP^-HA, tssA1_Nt1_^PA^tssA1_CTD_^PP^*, *tssA1_Nt1_^PP^tssA1_CTD_^PA^* anti-HA


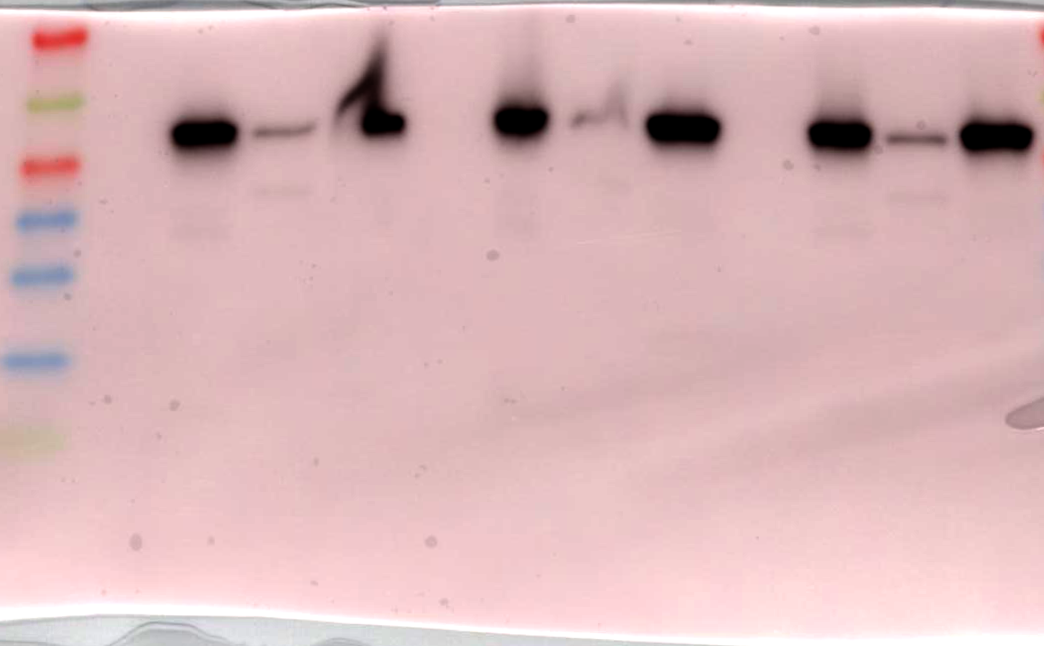


*tssA1^PP^-HA*, anti-RpoB


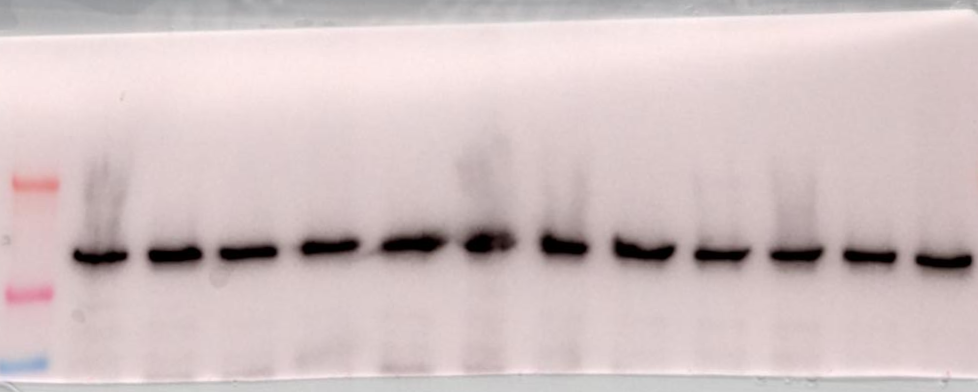

Supplement: Supplementary file 4 — Source data [file 41467_2024_48487_MOESM4_ESM.zip › Source Data/Source Data Supplementary Figure 7.docx]
