# Supplementary figures and images for: Functionality of chimeric TssA proteins in the type VI secretion system reveals sheath docking specificity within their N-terminal domains
Source: Nat Commun. 2024 May 20;15:4283. doi: 10.1038/s41467-024-48487-8 (PMC11106082; doi:10.1038/s41467-024-48487-8)

**Figure 4a**


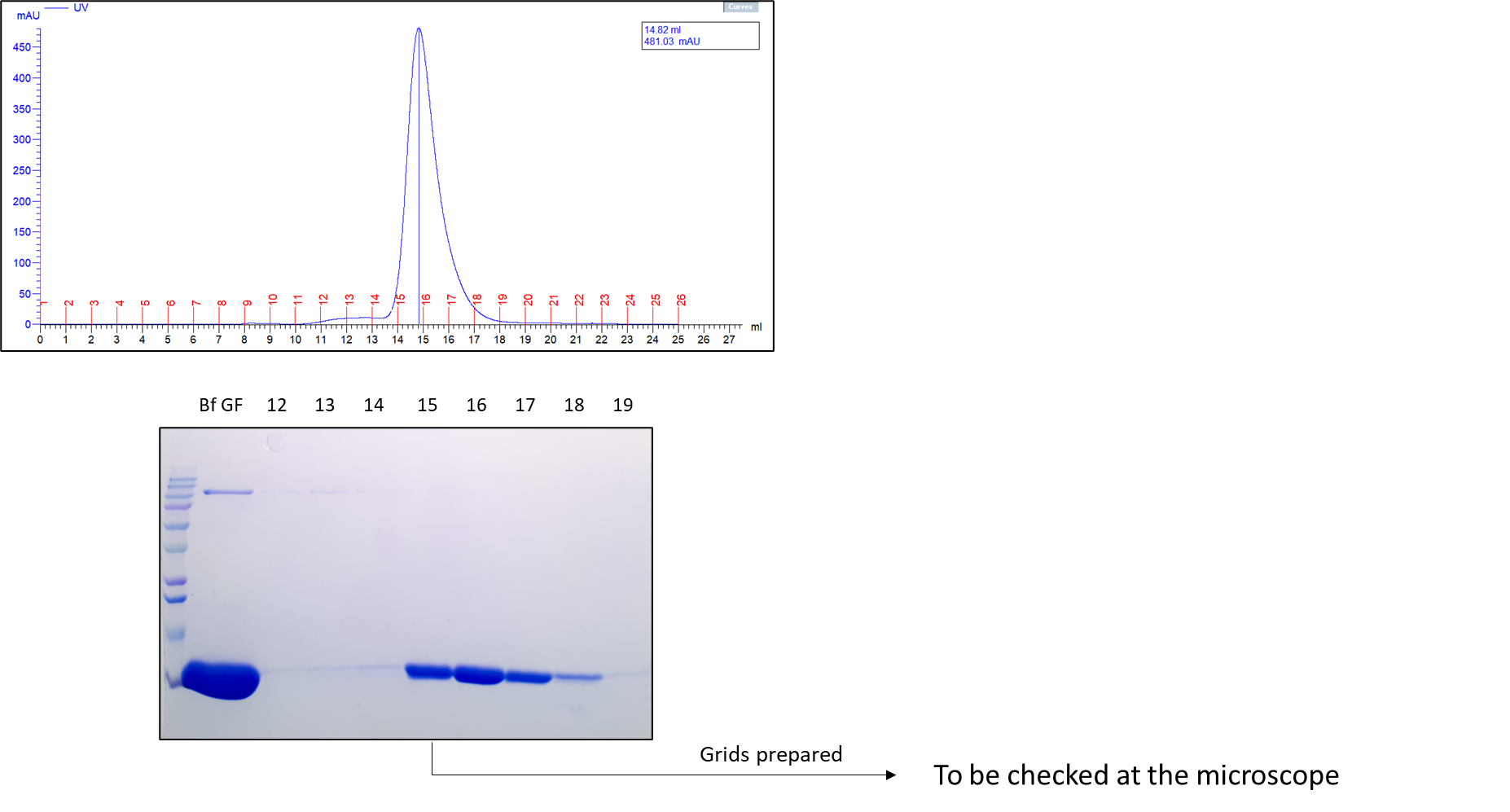


**Figure 4b**


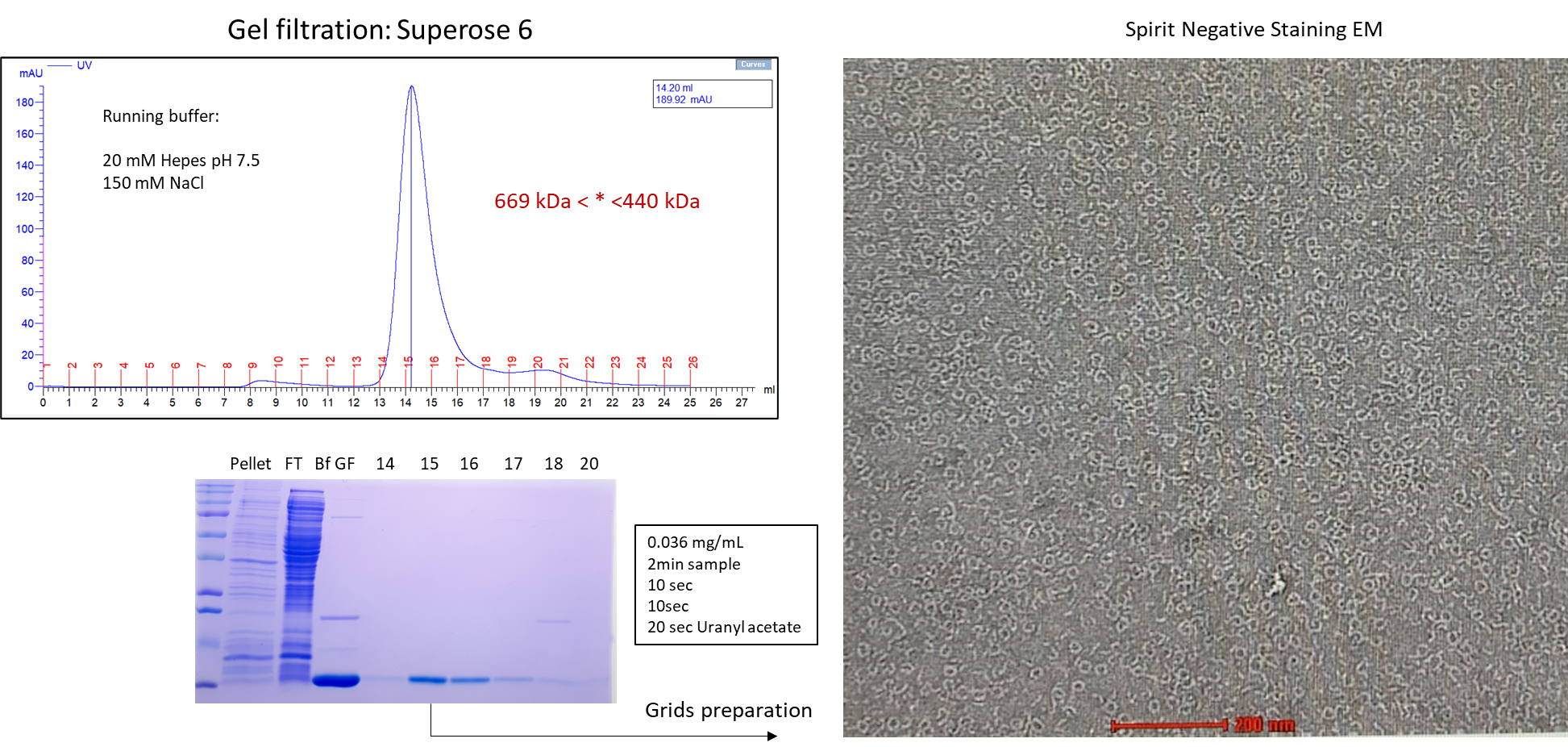

Supplement: Supplementary file 4 — Source data [file 41467_2024_48487_MOESM4_ESM.zip › Source Data/Source Data Figure 4.docx]

**Figure 7a**

Hcp2 cell


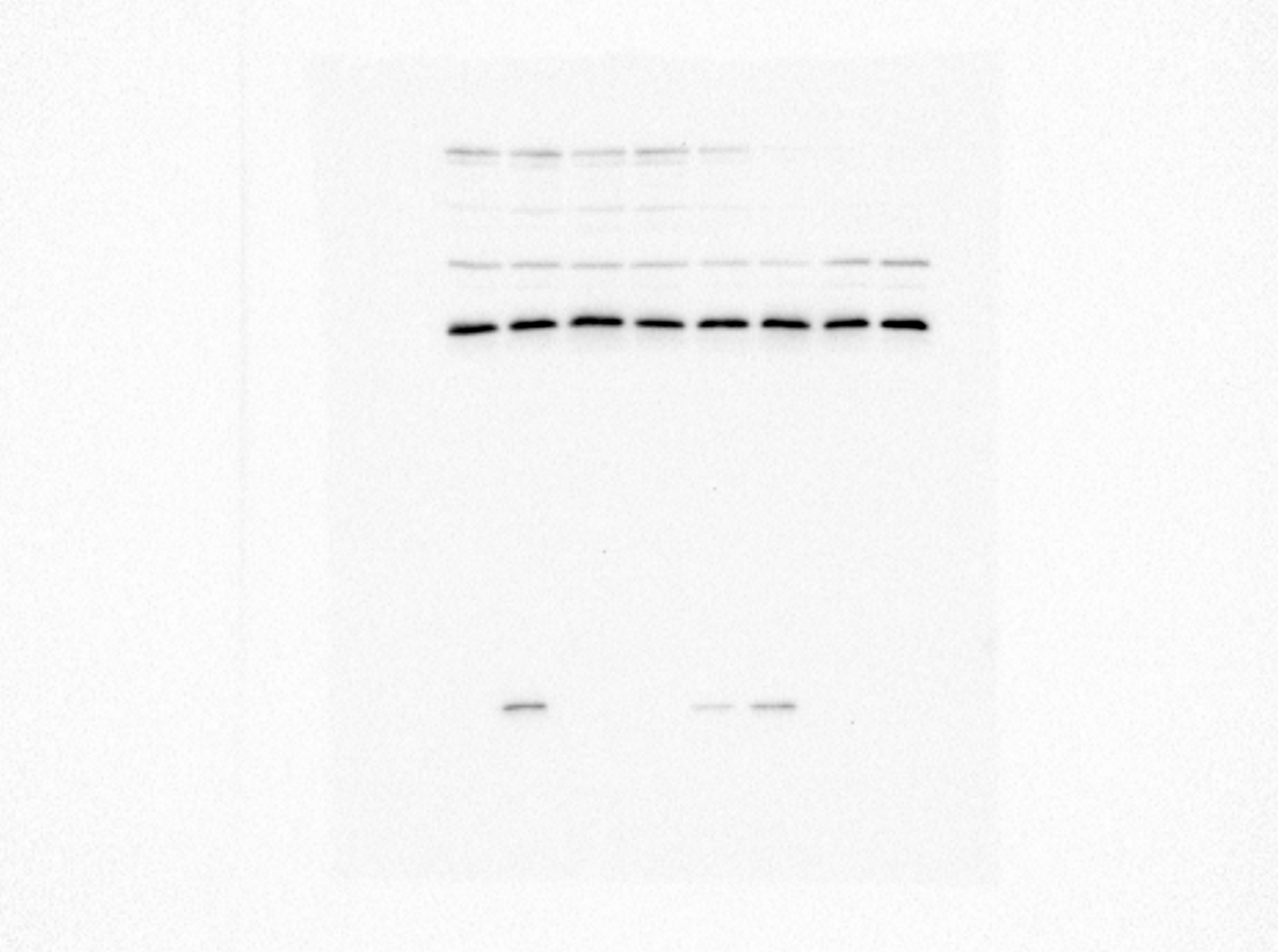


Hcp2 supernatant


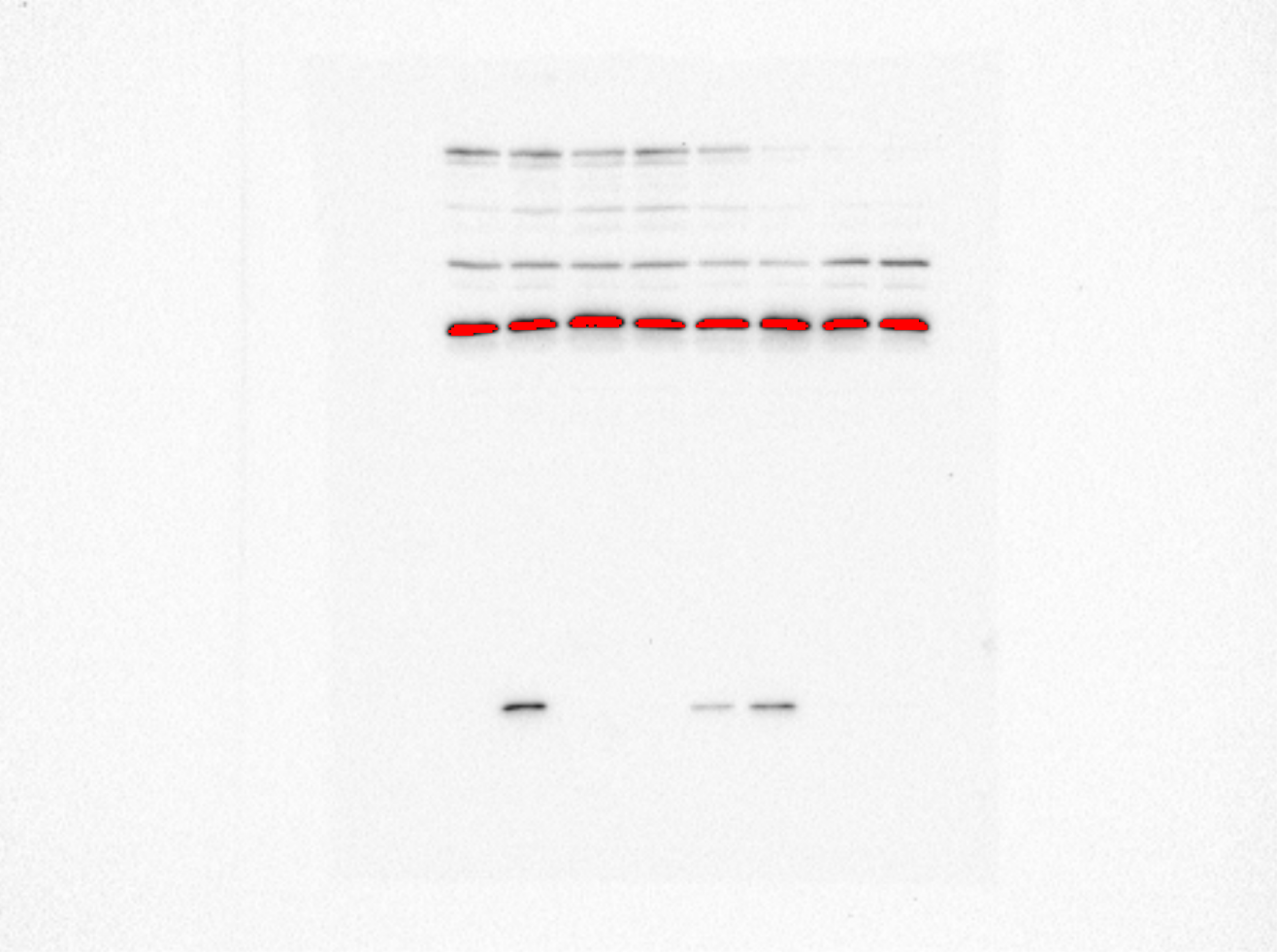


RpoB supernatant and cell


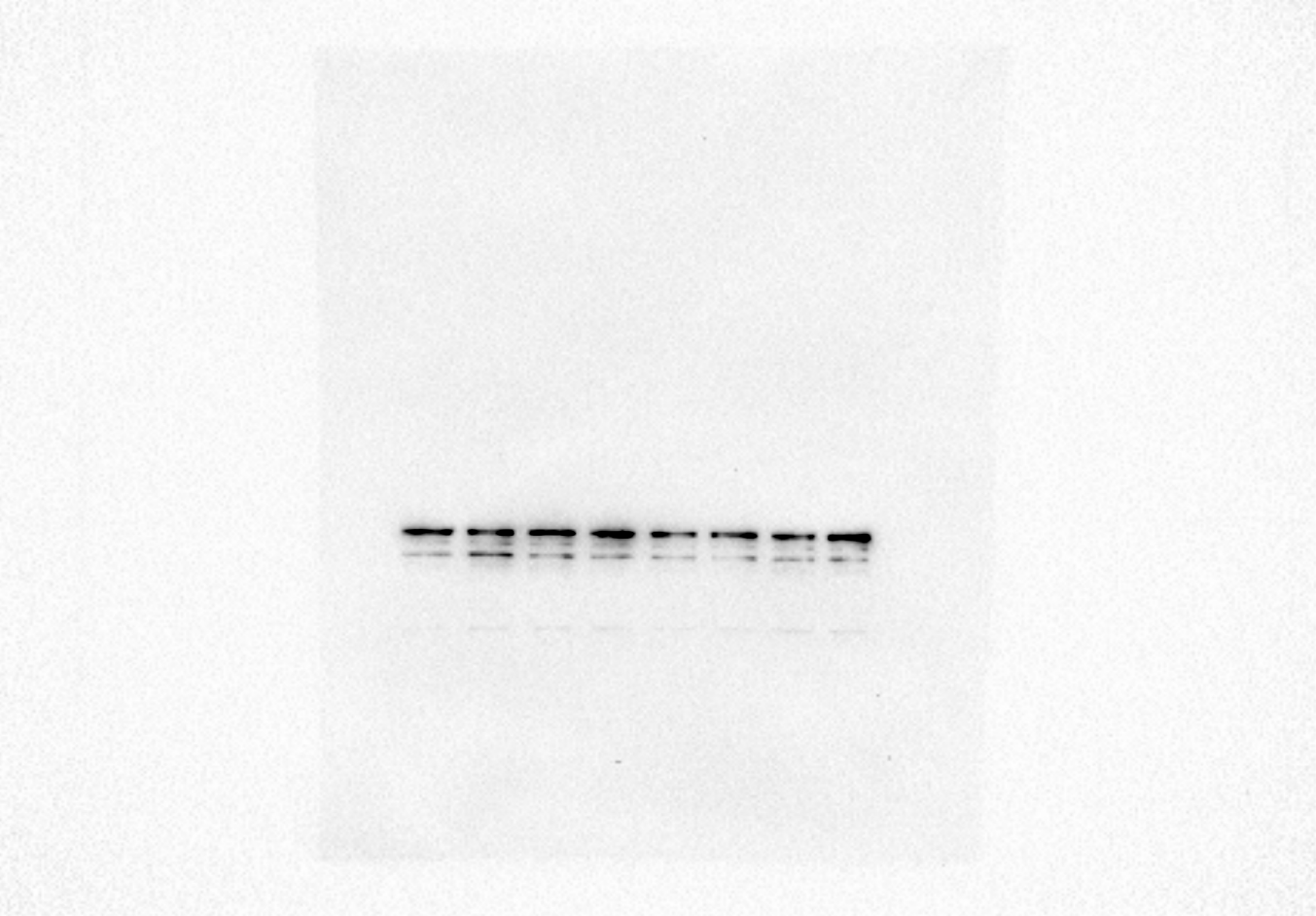

Supplement: Supplementary file 4 — Source data [file 41467_2024_48487_MOESM4_ESM.zip › Source Data/Source Data Figure 7.docx]

**Figure 8e**

Hcp1 cell and supernatant


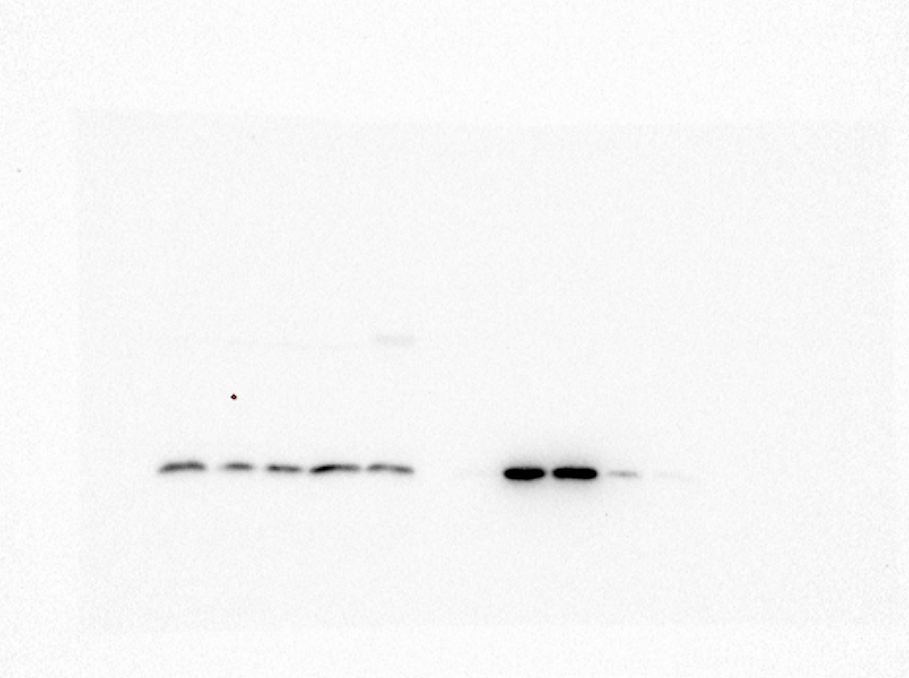


RpoB cell and supernatant


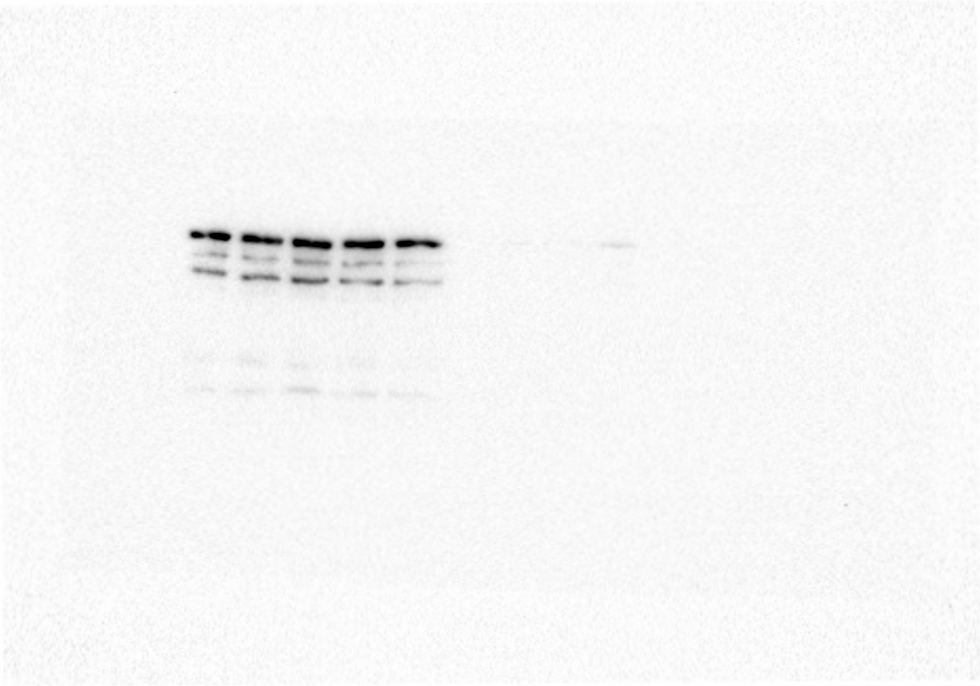

Supplement: Supplementary file 4 — Source data [file 41467_2024_48487_MOESM4_ESM.zip › Source Data/Source Data Figure 8.docx]

**Supplementary Figure 2a**

TssA1 anti-HA


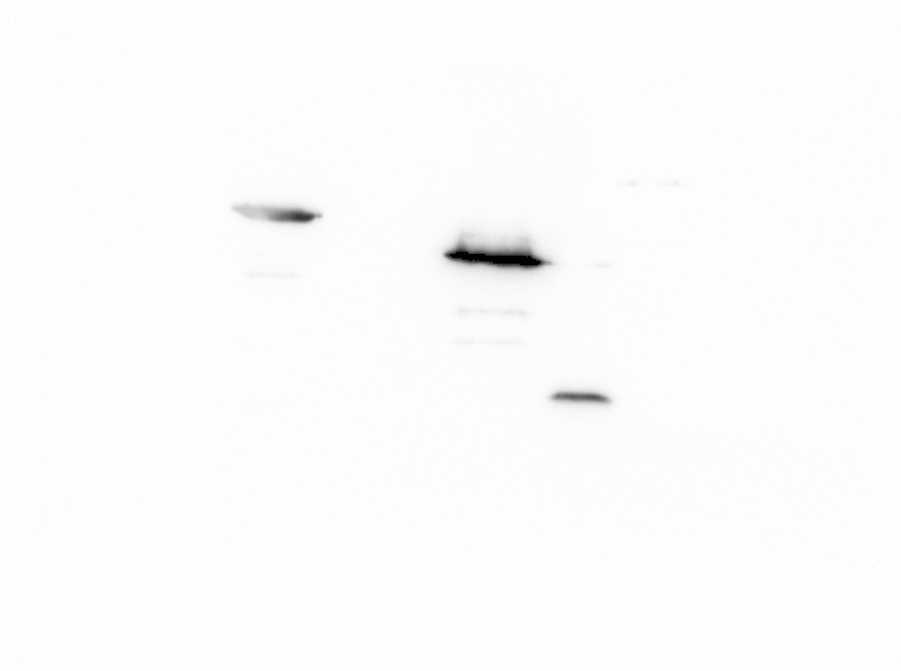


TssA2 anti-HA


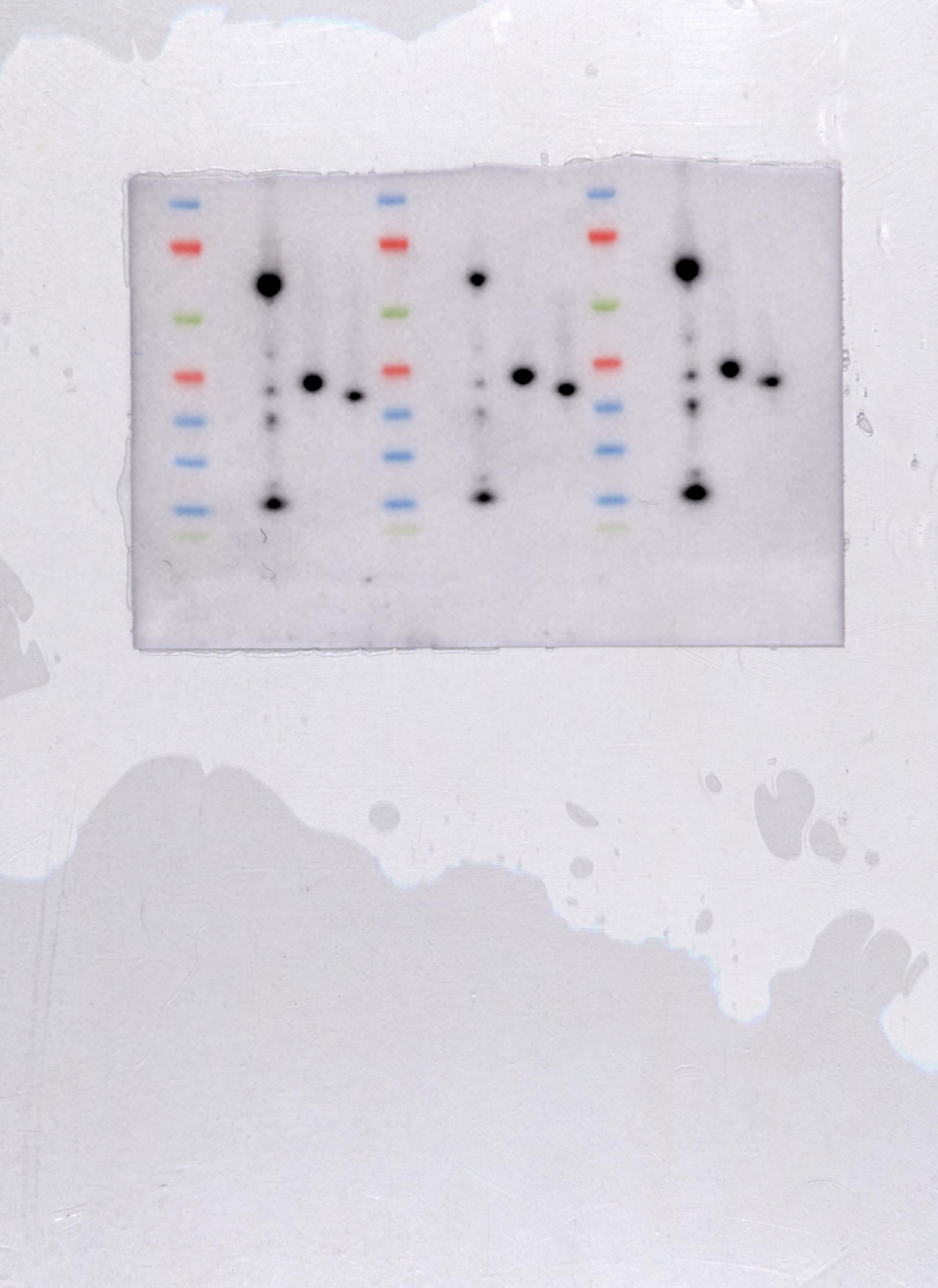


TssA2 anti-RpoB


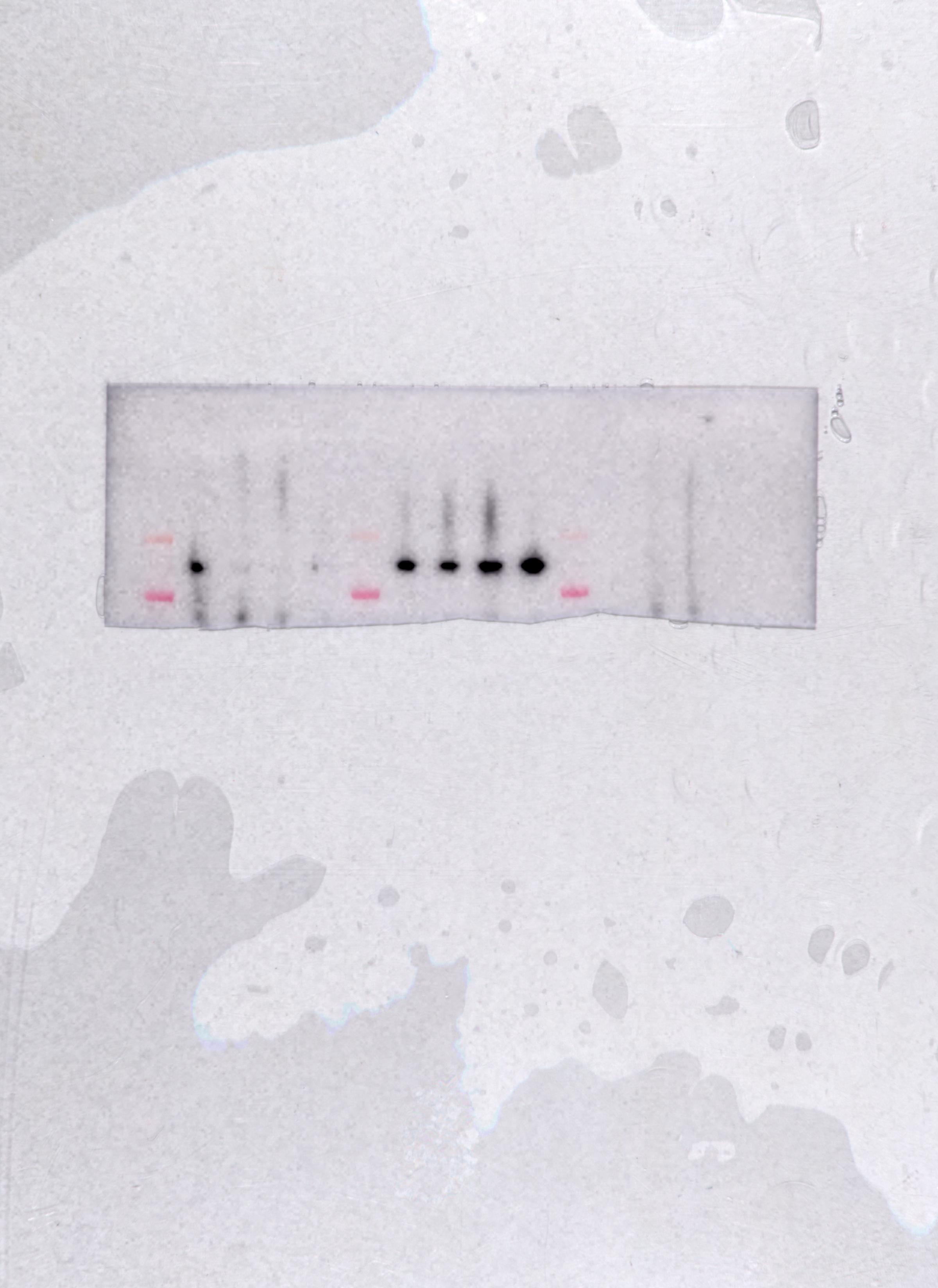


TssA3 anti-HA

TssA3 anti-RpoB

Supplement: Supplementary file 4 — Source data [file 41467_2024_48487_MOESM4_ESM.zip › Source Data/Source Data Supplementary Figure 2.docx]

**Supplementary Figure 5**


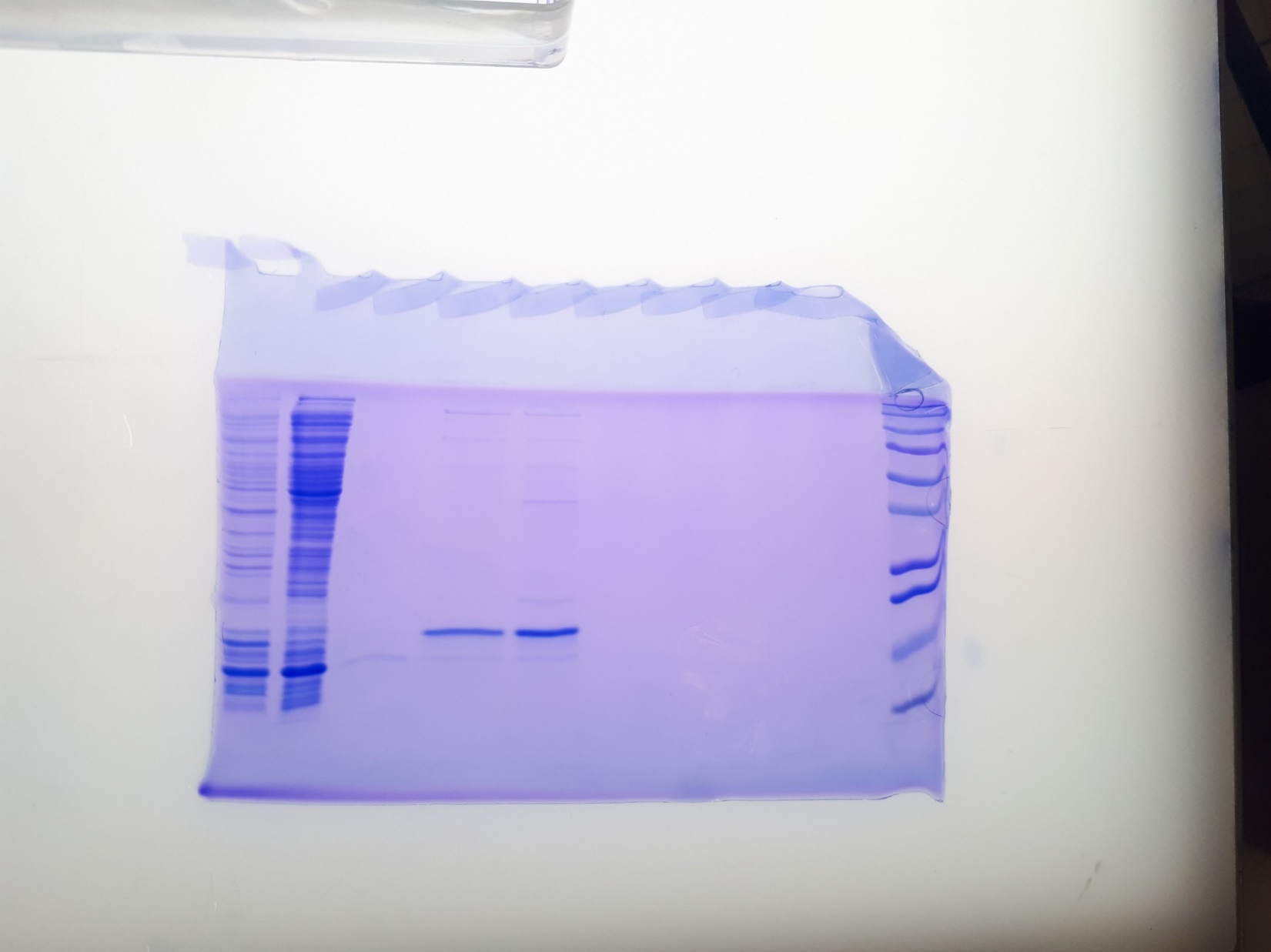

Supplement: Supplementary file 4 — Source data [file 41467_2024_48487_MOESM4_ESM.zip › Source Data/Source Data Supplementary Figure 5.docx]

**Supplementary Figure 9c**

Anti-HA


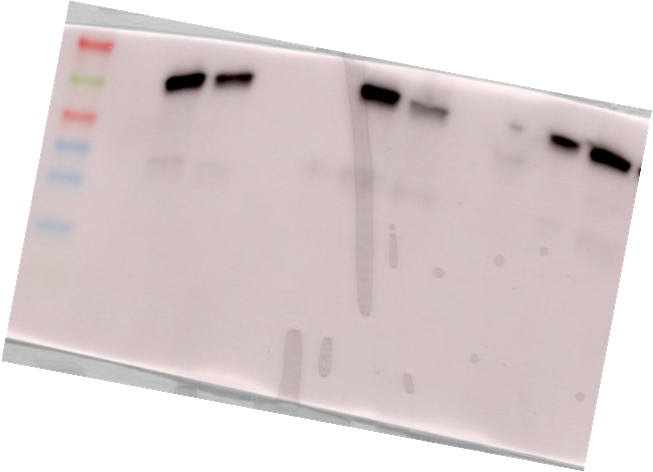


Anti-RpoB


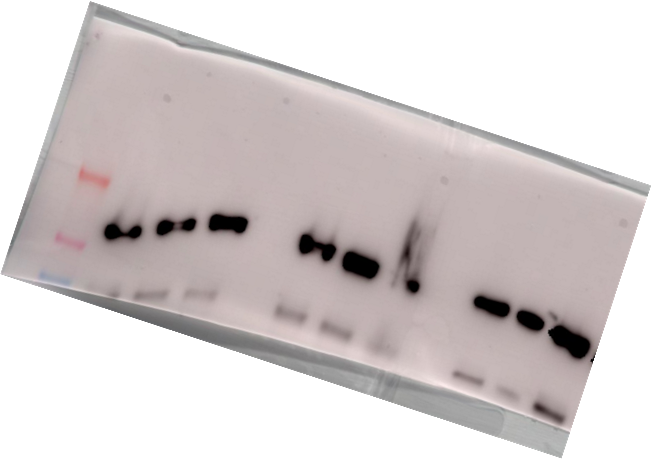

Supplement: Supplementary file 4 — Source data [file 41467_2024_48487_MOESM4_ESM.zip › Source Data/Source Data Supplementary Figure 9.docx]
